# Comparative analysis of *Porphyromonas gingivalis* A7436 and ATCC 33277 strains reveals differences in the expression of heme acquisition systems

**DOI:** 10.1128/spectrum.02865-23

**Published:** 2024-01-30

**Authors:** Michał Śmiga, Paulina Ślęzak, Teresa Olczak

**Affiliations:** 1 Laboratory of Medical Biology, Faculty of Biotechnology, University of Wroclaw, Wroclaw, Poland; Griffith University - Gold Coast Campus, Australia

**Keywords:** *Porphyromonas gingivalis*, strain variation, HmuY, IhtB, gingipain, heme, iron, hemoglobin, heme acquisition, virulence

## Abstract

*Porphyromonas gingivalis* strains exhibit different phenotypes *in vitro*, different virulence potential in animal models, and different associations with human diseases, with strains classified as virulent/more virulent (e.g., A7436 and W83) or as less virulent/avirulent (e.g., ATCC 33277). In this study, we comparatively analyzed the A7436 and ATCC 33277 strains to better understand their variability. Global gene expression analysis in response to heme and iron limitation revealed more pronounced differences in the A7436 than in the ATCC 33277 strain; however, in both strains, the largest changes were observed in genes encoding hypothetical proteins, genes whose products participate in energy metabolism, and in genes encoding proteins engaged in transport and binding proteins. Our results confirmed that variability between *P. gingivalis* strains is due to differences in the arrangement of their genomes. Analysis of gene expression of heme acquisition systems demonstrated that not only the availability of iron and heme in the external environment but also the ability to store iron intracellularly can influence the *P. gingivalis* phenotype. Therefore, we assume that differences in virulence potential may also be due to differences in the production of systems involved in iron and heme acquisition, mainly the Hmu system. In addition, our study showed that hemoglobin, in a concentration-dependent manner, differentially influences the virulence potential of *P. gingivalis* strains. We conclude that iron and heme homeostasis may add to the variability observed between *P. gingivalis* strains.

**IMPORTANCE:**

Periodontitis belongs to a group of multifactorial diseases, characterized by inflammation and destruction of tooth-supporting tissues. *P. gingivalis* is one of the most important microbial factors involved in the initiation and progression of periodontitis. To survive in the host, the bacterium must acquire heme as a source of iron and protoporphyrin IX. *P. gingivalis* strains respond differently to changing iron and heme concentrations, which may be due to differences in the expression of systems involved in iron and heme acquisition. The ability to accumulate iron intracellularly, being different in more and less virulent *P. gingivalis* strains, may influence their phenotypes, production of virulence factors (including proteins engaged in heme acquisition), and virulence potential of this bacterium.

## INTRODUCTION

Periodontal diseases belong to a group of multifactorial diseases, initiated by an ecological shift in the composition of oral biofilm and an exaggerated host immune response, which results in inflammation and destruction of tooth-supporting tissues, and often in tooth loss (1–5). Several articles have reported that a significant percentage of the human population is characterized either by a moderate or a severe form of periodontitis (6–13). *Porphyromonas gingivalis* is considered the main etiological agent and keystone pathogen responsible for dysbiosis in the oral microbiome, subsequent development of periodontitis, and contribution to systemic diseases (1, 14–16). Although the bacterium resides in the oral cavity of healthy individuals (10%–25%), it has been found in increased numbers in individuals with periodontitis (79-90%) (17–20).

Many studies have shown that *P. gingivalis* strains exhibit different phenotypes and pathogenicity, which are caused mainly by variations in capsular polysaccharides, the major and minor fimbriae, adhesion domains of lysine-specific gingipain (Kgp) and hemagglutinin A (HagA) (21–43). Although low sequence divergence within genes encoding catalytic domains of gingipains has been found (21), *P. gingivalis* clinical isolates exhibited different rates and distribution of Kgp and arginine-specific gingipain (RgpA and RgpB) activities (23). Sequencing of several *P. gingivalis* genomes revealed that the bacterium can exchange chromosomal DNA by specific gene rearrangements and allelic exchange through horizontal gene transfer using natural transformation and conjugation (20, 21, 44–46). Importantly, the spontaneous generation of phenotypically different sub-strains, caused in part by differences in genes encoding hemagglutinin domains of Kgp and HagA, can also occur, resulting in significant attenuation in a mouse virulence model (47–50). On the other hand, many genes are highly conserved in *P. gingivalis* strains and located in the core genome (22), including genes encoding heme acquisition Hmu and Iht systems (22, 51–53). The best characterized, and important for *P. gingivalis* virulence, is a heme uptake (Hmu) system composed of HmuY (a heme-binding hemophore-like protein), HmuR (a typical TonB-dependent receptor transporting heme through the outer membrane), and four proteins with unknown functions (HmuSTUV) (52, 54, 55). The Hmu system works in synergy with gingipains and their production is correlated (55–57). Apart from nutrient acquisition for this asaccharolytic pathogen and for avoiding the host immune response (58), gingipains participate in heme acquisition by RgpA-dependent oxidation of oxyhemoglobin to methemoglobin, allowing direct heme sequestration by HmuY (57) or heme release by proteolytic digestion of methemoglobin, the latter activity carried out mainly by Kgp (58, 59). Another *P. gingivalis* system that is considered to be an alternative one involved in heme and/or iron uptake is the Iht system (53); however, it is hardly characterized. The Iht system comprises the TonB-dependent outer membrane receptor (IhtA), putative heme-binding lipoprotein (IhtB), periplasmic binding protein (IhtC), permease (IhtD), and cytoplasmic ATP binding protein (IhtE). Due to IhtB homology to CbiK chelatase, it is thought that IhtB could remove iron from heme and transfer it to the IhtA protein for subsequent iron transfer to the periplasm. Iron transport to the cytoplasm could be performed by IhtC-E proteins (55, 60).

*P. gingivalis* does not synthesize heme *de novo*, and consequently it produces numerous heme acquisition systems, allowing this bacterium to efficiently take up heme, as a source of both iron and protoporphyrin IX (PPIX), to survive inside the host and initiate the pathogenic process. Interestingly, it has been shown that *P. gingivalis* strains respond differently to changing iron and heme concentrations in the external environment (61). Therefore, we believe that differences in the virulence potential of *P. gingivalis* strains selected in this study may also be due to differences in the expression of systems involved in iron and heme acquisition. To analyze differences between *P. gingivalis* strains in their phenotypes and response to iron and heme starvation, we comparatively examined the less virulent, non-capsulated, highly fimbriated ATCC 33277 (33277) strain and more virulent, capsulated, poorly fimbriated A7436 and W83 strains.

## RESULTS

### Differences in genome organization of *P. gingivalis* strains

**Fig 1:**
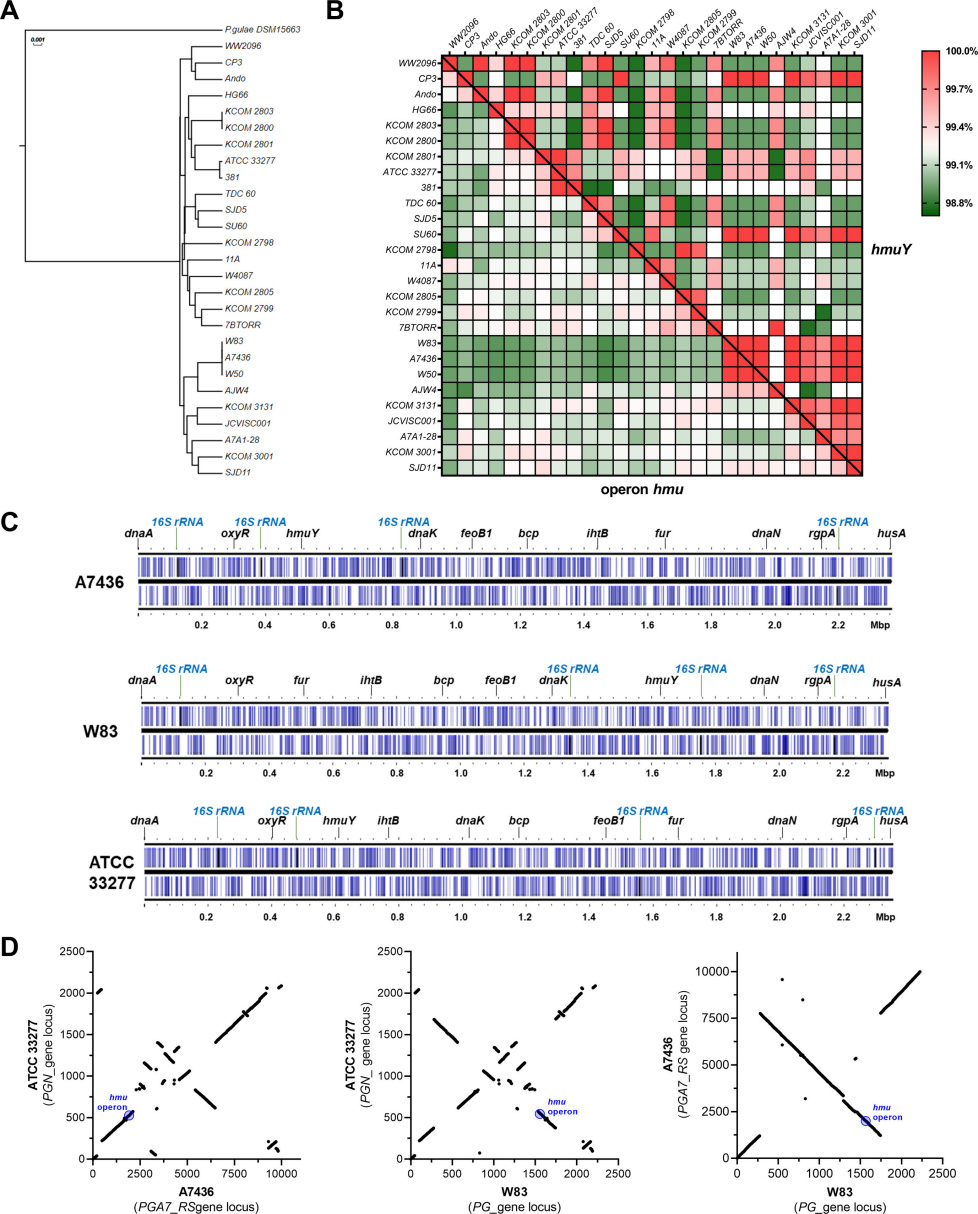
Comparative analysis of selected genomes of *P. gingivalis* strains. (**A**) A phylogenetic tree was constructed based on nucleotide sequences of the *hmu* operon present in strains listed in Table 1. Sequence for *P. gulae* (DSM15663 strain) was used as an out-group. (**B**) A heat map was constructed based on nucleotide sequences of the *hmuY* gene and nucleotide sequences of the entire *hmu* operon. The color gradient from green to red shows the percentage of identity from lowest (green) to highest (red). (**C**) Linearized genome maps of *P. gingivalis* A7436, ATCC 33277, and W83 strains demonstrating the location of selected genes. The genomes were visualized with Proksee (https://proksee.ca/). (**D**) Graphical presentation of the arrangement of genes in pairs of selected genomes. The *hmu* operon location is marked on the graphs as a reference. The changes in the slope of the curve represent the inversion of chromosome fragments of the strain indicated at the Y-axis in comparison to the reference strain at the X-axis.

Analysis of known *P. gingivalis* genomes revealed significant differences among laboratory strains and clinical isolates. According to the NCBI database (www.ncbi.nlm.nih.gov; as of June 26, 2023), over 90 *P*. *gingivalis* genomes have been fully or partially sequenced, and some examples of representative genomes are listed in Table 1; Fig. 1A. To perform a detailed analysis, we chose laboratory strains used in our previous studies (A7436 and 33277), and in addition, in some experiments, a reference W83 strain was employed. First, we constructed a phylogenetic tree based on nucleotide sequences of the *hmu* operon of selected strains (Fig. 1A). In contrast to the high homology of the *hmuY* gene, other genes of the *hmu* operon exhibited lower homology (Fig. 1B), thus confirming the high significance of HmuY protein. Furthermore, we analyzed the location of selected genes responsible for iron and heme uptake, gene regulation, and replication. Differences in their location in respective strains (Fig. 1C) might result from chromosome inversions and insertions (Fig. 1D). Moreover, our comparative analysis of A7436, 33277, and W83 genomes showed differences in the *hmu* operon location in relation to other genes (Fig. 1C and D), which might result mostly from chromosome rearrangements.

**TABLE 1:** Genome and *hmu* operon access IDs of selected *P. gingivalis* strains

Strain	GenBank sequence ID	*hmu* operon locus ID (*hmuY-hmuV*)
381	CP012889.1	PGF_00005370 – PGF_00005320
11A	FUFE01000003.1	B0490_RS00365 – B0490_RS00390
7BTORR	FUFD01000063.1	PGIN_7BTORR_01725 – PGIN_7BTORR_01720
A7436	CP011995.1	PGA7_RS02055 – PGA7_RS02030
A7A1-28	CP013131.1	PGS_00005310 – PGS_00005360
AJW4	CP011996.1	PGJ_00004400 – PGJ_00004350
Ando	BCBV01000036.1	PGANDO_0790 – PGANDO_0795
ATCC 33277	CP025930.1	PGN_0558 – PGN_0553
CP3	SGBA01000005.1	EW639_02390 – EW639_02415
HG66	CP007756.1	EG14_03640 – EG14_03615
JCVI SC001	APMB01000180.1	A343_2305 – A343_2311
KCOM 2798	CP024598.1	CS374_01645 – CS374_01670
KCOM 2799	CP024601.1	CS387_10190 – CS387_10165
KCOM 2800	CP024599.1	CS388_02390 – CS388_02415
KCOM 2801	CP024600.1	CS543_09515 – CS543_09540
KCOM 2803	CP024592.1	CS545_07860 – CS545_07885
KCOM 2805	CP024594.1	CS548_03395 – CS548_03370
KCOM 3001	CP024595.1	CS550_06815 – CS550_06840
KCOM 3131	CP024596.1	CS549_07880 – CS549_07905
SJD11	ASYO01000134.1	SJDPG11_09125 – SJDPG11_09100
SJD5	ASYN01000124.1	SJDPG5_04955 – SJDPG5_04930
SU60	FUFI01000019.1	PGIN_YH522_00859 – PGIN_YH522_00864
TDC60	CP025931.1	CF002_0748 – CF002_0743
W4087	KI260273.1	HMPREF1990_02097 – HMPREF1990_02102
W50	CP092048.1	MCS25_07005 – MCS25_07030
W83	AE015924.1	PG1551 – PG1556
WW2096	NSLX01000036.1	CLI72_08345 – CLI72_0837

### Response to iron and heme limitation varies in *P. gingivalis* A7436 and 33277 strains

**Fig 2:**
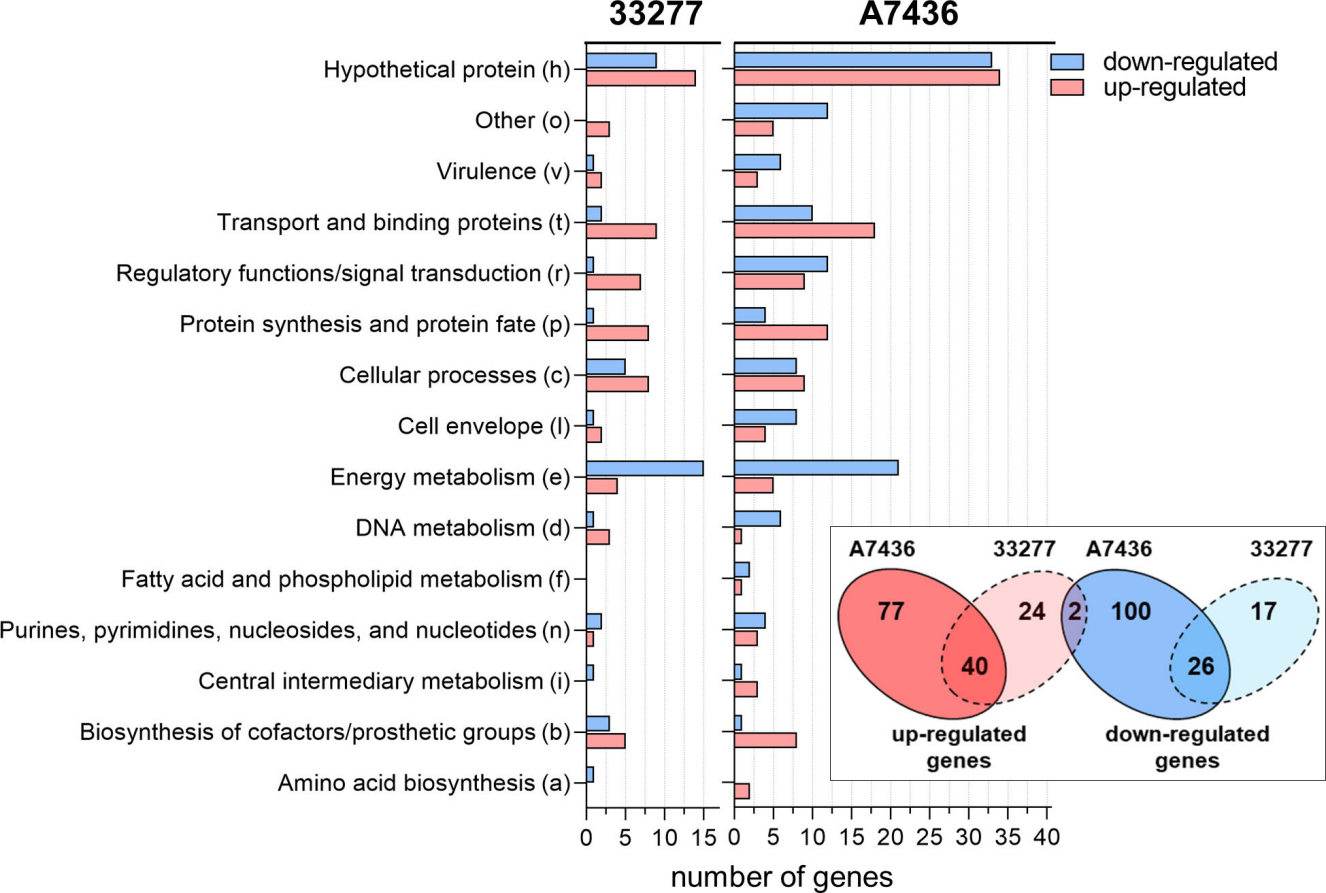
General summary of microarray data of gene expression in *P. gingivalis* A7436 and ATCC 33277 strains grown in iron- and heme-depleted conditions (DIP medium) in comparison to iron- and heme-replete conditions (Hm medium). Data are shown from three independent biological replicates. Up- and down-regulated genes are grouped according to their function. The inset shows the Venn diagram showing either the number of genes whose expression changed in both tested strains or the number of genes whose expression is strain-specific.

First, global gene expression analysis was carried out in *P. gingivalis* A7436 and 33277 strains. Relative differences in gene expression in bacteria grown in iron- and heme-depleted conditions (DIP medium) in relation to iron- and heme-replete conditions (Hm medium) were examined using microarray analysis. In the A7436 strain, the expression of 117 genes was up-regulated and the expression of 128 genes was down-regulated, whereas in the 33277 strain changes in gene expression of the lower number of genes were observed (43 and 66 genes, respectively). The most pronounced differences were found in genes belonging to the group of hypothetical proteins, in genes whose products participate in energy metabolism, and in genes encoding proteins engaged in transport and binding (Fig. 2). Proteins ascribed to those groups are listed in Tables S1 and S2. It is also worth noting here that although the microarray analysis was designed based on the A7436 genome, which could result in no detection of changes in the expression of genes specific to the 33277 strain only, genes important for *P. gingivalis* virulence are located in the core genome (22). Although the expression of a different number of genes was changed in A7436 and 33277 strains, the expression of the same 40 genes was up-regulated under iron and heme starvation in both examined strains (Fig. 2). Among them are genes encoding proteins responsible for iron and heme uptake and their homeostasis (e.g., Hmu system, FeoB, Dps), oxidative stress response (e.g., AhpC, AhpF, flavodoxin), transport (e.g., TonB-dependent receptors, ABC transporters, metal transporters, TolC-like protein), regulation and signal transduction (e.g., FeoA, PorX, LuxR, SigH) (Table S1). Interestingly, changes in the expression of many genes were strain specific but less pronounced (Fig. 2; Table S1). In the A7436 strain cultured under iron and heme starvation, increased expression of genes encoding some proteases, including *rgpB* (FC ~2.5×), *PG1788* (FC ~2.2×), *PG1855* (FC ~2.7×), and *PG2029* (FC ~2.1×) genes, as well as genes encoding proteins involved in transport, including membrane-associated heme-binding protein HBP35 (FC ~2.5×), type IX secretion system (T9SS) protein PorS (FC ~3.3×), putative sulfur exporter (PG2004; FC ~2.6×), and CorA family protein (FC ~3.2×), was detected. Surprisingly, only in the A7436 strain was an expression of the gene encoding HmuT protein changed (FC ~57.1×), while the expression of the rest of the genes of the *hmu* operon increased in both strains. Genes whose expression increased only in the 33277 strain include mostly ones encoding hypothetical proteins and ribosomal proteins (e.g., *rsmH*, *rprR*, *rpmI* genes).

Greater discrepancies between A7436 and 33277 strains in gene expression were detected in genes whose expression decreased when bacteria were grown under iron and heme starvation (Table S2). Only 26 genes are common to tested strains whose expression decreased, being ~20% and ~60% of total genes whose expression decreased in A7436 and 33277 strains, respectively. They include mainly genes encoding proteins involved in energy metabolism (e.g., oxidoreductases, ferredoxin, electron transport complex proteins). The greatest decrease in gene expression, especially in the 33277 strain (FC ~−13.3× and ~ −61.2× in A7436 and 33277 strains, respectively), was observed for a gene encoding a protein from the rubrerythrin family (PG0195), which is employed in the response to oxidative stress (62, 63). Decreased gene expression only for the A7436 strain included genes encoding proteins involved in energy metabolism (e.g., cytochrome *c* biogenesis proteins, IspH, TpiA), transport (e.g., HusA, HusB, PorT), and cell envelope and virulence (e.g., WecC, PG0109, PG0411—hemagglutinin, LptC, FimA, FimB). On the other hand, the expression of only a few genes changed specifically in the 33277 strain under iron and heme starvation, including genes encoding hemolysin secretion protein D (PG0093; FC ~−2.1×), ferredoxin (FC ~−3.0×), HemG (FC ~−2.8×), and GltA (FC ~−2.9×). In addition, the expression of two genes (*PG2038* and *PG2040*) decreased (FC for both ~−2.2×) and increased (FC for both ~2.5×) in A7436 and 33277 strains, respectively, in response to iron and heme limitation (Tables S1 and S2). The two genes seem to be co-transcribed and encode *N*-acetylmuramoyl-L-alanine amidase and histidinol phosphate phosphatase.

To validate the results obtained by microarray analysis, we examined the expression of selected genes using RT-qPCR in bacteria cultured for 24 hours (early stationary growth phase) under iron and heme starvation in comparison to iron- and heme-replete conditions (DIP medium vs Hm medium). Moreover, to understand in more detail the expression pattern of genes involved in iron and heme uptake, we also analyzed gene expression in bacteria cultured for 10 hours (late exponential growth phase). As shown in Table 2, the results obtained with RT-qPCR are in general consistent with the results obtained with microarray analysis. As our previous studies have shown (52, 64, 65), expression of the *hmuY* gene in the A7436 strain increased under iron and heme starvation. Furthermore, our analysis showed that the expression of *hmuY* and *hmuR* genes increased during prolonged growth in both A7436 and 33277 strains. A similar effect was observed for the *feoB* genes (PG1043 and PG1294), whose products are engaged in ferrous iron/manganese transport (66–69). In addition, the *feoB* gene (PG1043), encoded close to the *feoA* gene (PG1044), was shown to be engaged in protection from oxidative stress (67, 68). Expression of *ihtB*, *rgpA*, *rgpB,* and *kgp* genes changed throughout the culture time, with a decrease after 10 hours and an increase after 24 hours in the DIP medium in comparison to the Hm medium. Although the changes were more evident in the A7436 strain, they were not statistically significant for both examined strains.

**TABLE 2:** Gene expression analyzed using RT-qPCR. Relative transcript levels were determined in bacteria grown in iron- and heme-depleted conditions (DIP medium) in comparison to iron- and heme-replete conditions (Hm medium)

Gene name	W83	A7436			33277		
	Locus ID	Locus ID	Fold change		Locus ID	Fold change	
			10 hours	24 hours		10 hours	24 hours
*hmuY*	PG1551	PGA7_RS02055	90.38 ± 52.06	260.31 ± 189.67	PGN_0558	5.34 ± 0.75	125.37 ± 27.10
*hmuR*	PG1552	PGA7_RS02050	69.94 ± 35.30	266.43 ± 142.74	PGN_0557	4.43 ± 0.24	243.28 ± 75.77
*husA*	PG2227	PGA7_RS10010	−1.42 ± 0.17	−2.09 ± 0.57	PGN_2091	1.08 ± 0.27	1.26 ± 0.18
*ihtB*	PG0669	PGA7_RS06060	−2.18 ± 0.62	2.06 ± 0.37	PGN_0705	−1.16 ± 0.13	1.66 ± 0.06
*feoB*	PG1043	PGA7_RS04380	1.93 ± 1.03	3.53 ± 0.71	PGN_1309	1.90 ± 0.80	3.47 ± 0.54
*hagA*	PG1837	PGA7_RS08180	1.28 ± 0.54	−1.09 ± 0.37	PGN_1733	1.27 ± 0.45	1.51 ± 0.63
*kgp*	PG1844	PGA7_RS08195	−1.69 ± 1.32	3.90 ± 0.95	PGN_1728	1.16 ± 0.26	2.79 ± 0.83
*rgpA*	PG2024	PGA7_RS09040	−1.26 ± 0.71	4.24 ± 1.72	PGN_1970	−1.14 ± 0.25	4.52 ± 1.17
*rgpB*	PG0506	PGA7_RS06770	−1.90 ± 0.90	5.87 ± 4.37	PGN_1466	−1.35 ± 0.57	2.69 ± 0.49

### Main heme acquisition systems are expressed differently in A7436 and 33277 strains

**Fig 3:**
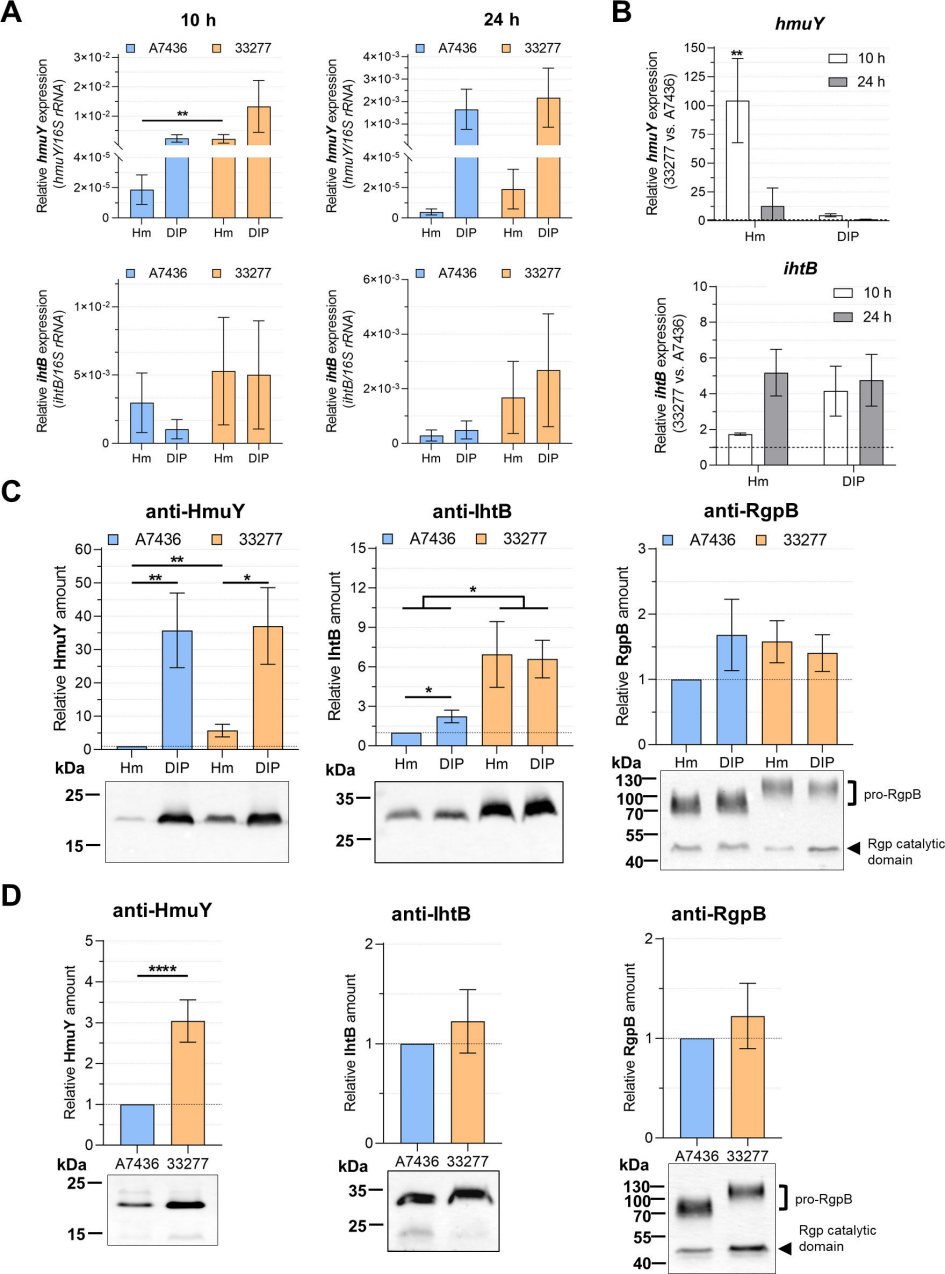
Analysis of production of selected proteins engaged in heme acquisition in *P. gingivalis*. RT-qPCR was used to determine differences in the expression of *hmuY* and *ihtB* genes during late exponential (10 hours; 10 h) and early stationary (24 hours; 24 h) growth phases. Results are shown as relative *hmuY* or *ihtB* mRNA levels in comparison to the *16S rRNA* gene (*hmuY/16S rRNA* or *ihtB/16S rRNA*) (**A**) or as expression fold change in the ATCC 33277 strain in comparison to the A7436 strain (33277 vs A7436) (**B**). Comparative analysis of cell-associated HmuY, IhtB, and RgpB proteins in *P. gingivalis* cultured in liquid media (**C**) and on anaerobic blood agar (ABA) plates (**D**) using Western blotting and densitometric analysis. Results were normalized to the A7436 strain grown in a liquid medium supplemented with iron and heme (**C**) or results are shown as relative protein production fold change in the ATCC 33277 strain in comparison to the A7436 strain (**D**). The results are shown as mean ± standard error (mean ± SE). *P < 0.05, **P < 0.01, ****P < 0.0001.

Genetic differences and different responses to iron and heme starvation between A7436 and 33277 strains have led us to compare the production of selected proteins involved in iron and heme uptake by *P. gingivalis*. First, we determined the expression of *hmuY* and *ihtB* genes in bacteria grown in different conditions (Fig. 3A). As expected, the transcript encoding the HmuY protein significantly increased during incubation in both A7436 and 33277 strains. Although expression of the *ihtB* gene was higher in the 33277 strain in the DIP medium and after 24 hours in the Hm medium, the observed differences were not statistically significant. Then, we compared transcript levels of *hmuY* and *ihtB* genes in the 33277 strain in comparison to the A7436 strain (Fig. 3B). We observed ~100× higher *hmuY* transcript production in the 33277 strain, as compared to the A7436 strain, after 10 hours in bacteria grown in iron- and heme-replete conditions (Hm medium). After 24 hours, expression of the *hmuY* gene did not differ significantly between the strains in bacteria cultured in both conditions (Hm and DIP media).

Next, using Western blotting with anti-HmuY and anti-IhtB antibodies, we analyzed the production of HmuY and IhtB proteins (Fig. 3C; Fig. S1). We showed that HmuY protein production was ~6× higher in the 33277 strain in comparison to the A7436 strain when culturing bacteria in iron-/heme-replete conditions, whereas the production of HmuY protein in the DIP medium was the same in both tested strains. The amount of IhtB protein was ~2× higher in the A7436 strain when bacteria were grown in the DIP medium, which is consistent with *ihtB* transcript production after 24 hours (Table 2). On the other hand, we did not observe differences in IhtB protein production in the 33277 strain between DIP and Hm media. However, we found ~2–6× higher IhtB protein production in the 33277 strain in comparison to the A7436 strain (Fig. 3C; Fig. S1).

**Fig 4:**
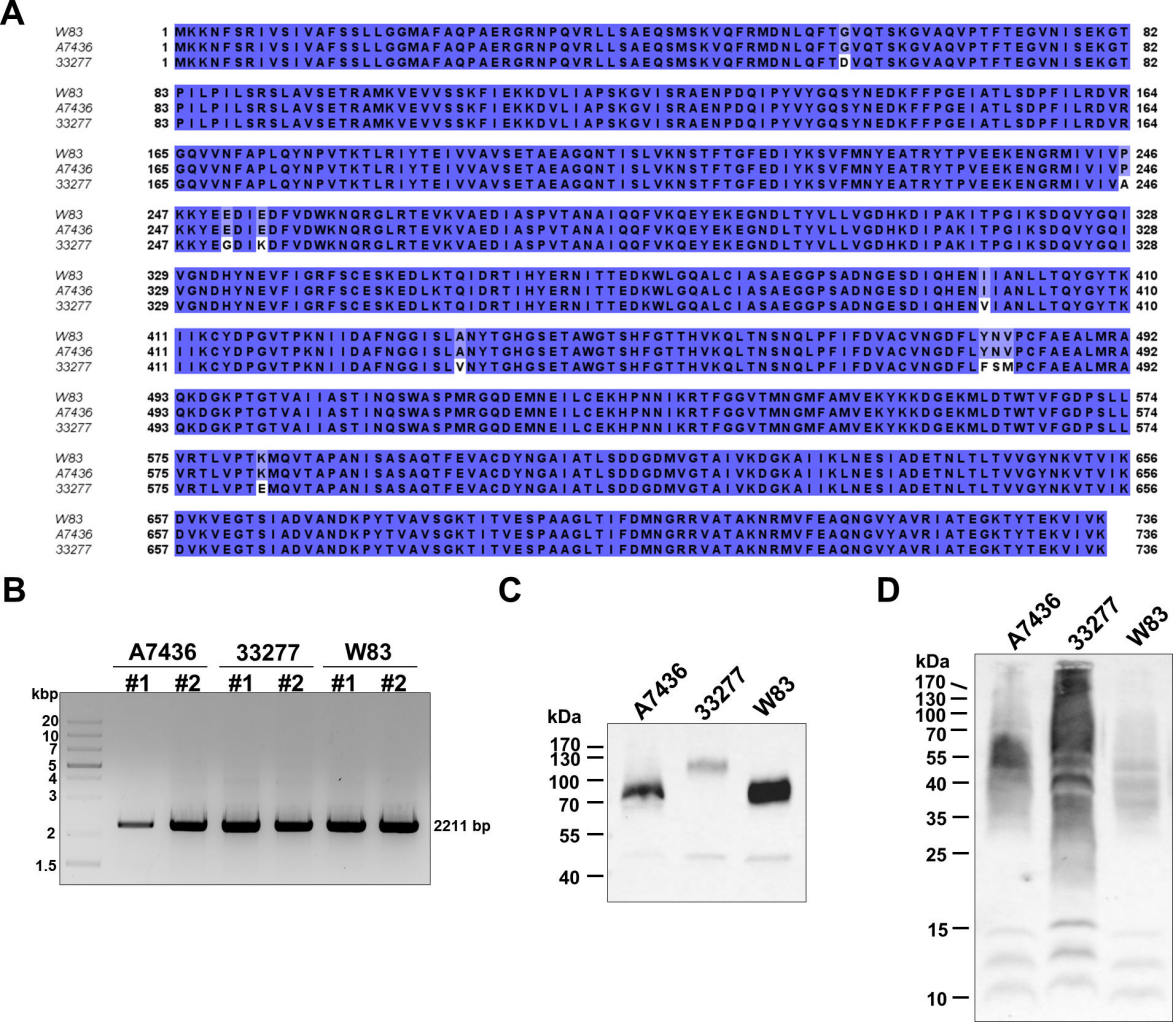
Comparison of RgpB produced by *P. gingivalis* A7436, 33277, and W83 strains. (**A**) Amino acid sequence alignment. (**B**) Gene length analysis. The whole DNA sequence of the *rgpB* gene was amplified using genomic DNA of A7436, 33277, or W83 strains and primers listed in Table S4. (**C**) Differences in RgpB molecular weight were examined in whole cell lysates by sodium dodecyl sulfate-polyacrylamide gel electrophoresis and Western blotting using anti-RgpB antibody. (**D**) Differences in the production of glycans were examined using reactivity with JACALIN (the lectin preferentially recognizes galactose linked with β−1,3 linkage to *N*-acetylgalactosamine and mono- or di-sialylated form of this structure).

Using microarray analysis, we detected an increase in *rgpB* expression (Table S1) in the A7436 strain when bacteria had been starved of iron and heme. Moreover, we found that expression of all genes encoding gingipains increased after 24 hours in iron- and heme-depleted conditions (Table 2). No differences in both conditions and between the examined strains were found in the expression of the *hagA*, the gene encoding the main hemagglutinin A (HagA), of which hemagglutinin/adhesion domains are present in RgpA and RgpB (Table 2). Therefore, we decided to analyze *rgpB* gene expression at the protein level. Expression of the cell-associated RgpB (70, 71), gingipain produced without hemagglutinin/adhesion domains, was similar in both examined strains (Fig. 3C and D; Fig. S1 and S2). However, some differences could be observed in the case of pro-RgpB protein associated with the outer membrane (Fig. 3C, D, and 4C), although neither *rgpB* gene length, amino acid sequence, nor theoretical RgpB molecular weight significantly differed in the examined strains (Fig. 4A and B).

To present in more detail differences in the production of HmuY, IhtB, and RgpB proteins in A7436 and 33277 strains, we tested protein levels in bacteria cultured on blood agar (ABA) plates, containing hemoglobin as the main heme source, and serum heme-binding proteins, mainly albumin. These conditions also allow *P. gingivalis* to deposit excess heme on the cell surface, providing the bacterium with the ability to maintain iron and heme homeostasis, similar to that found in the natural environment in advanced stages of the disease. As shown in Fig. 3D; Fig. S2, HmuY protein production was ~3× higher in the 33277 strain in comparison to the A7436 strain, whereas production of both IhtB and RgpB did not differ between examined strains. Our data demonstrate that production of the main components of heme acquisition systems may vary depending on the strain, therefore revealing bases of additional differences between *P. gingivalis* strains.

### Intracellular iron content influences *P. gingivalis* phenotype

**Fig 5:**
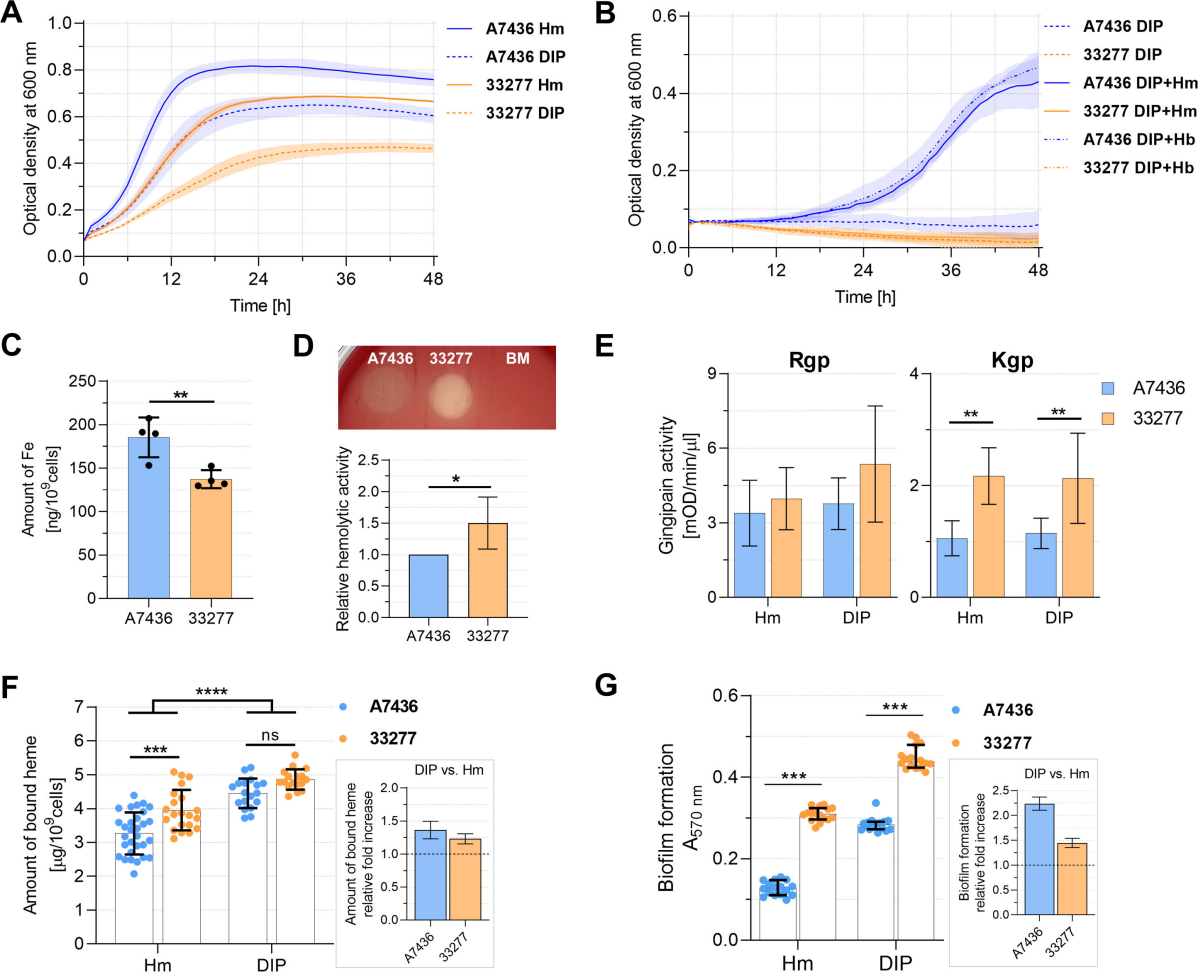
Phenotypic analysis of *P. gingivalis* A7436 and ATCC 33277 (33277) strains. (**A**) Growth of bacteria in liquid culture media containing iron and heme (iron- and heme-replete conditions, Hm medium) or without heme and supplemented with the iron chelator 2,2-dipyridyl (iron- and heme-depleted conditions, DIP medium). (**B**) Recovery of bacteria after prior heme and iron starvation. Bacteria were cultured for two passages in a DIP medium and then transferred to a fresh DIP medium supplemented with heme (DIP + Hm) or hemoglobin (DIP + Hb). Growth without an iron source was used as a control (DIP medium). Bacterial growth was monitored over time by measuring the optical density at 600 nm (statistical analysis is presented in Table S3). (**C**) The intracellular amount of iron was measured using a ferrozine-based assay. (**D**) Hemolytic activity was examined on anaerobic blood agar (ABA) plates after 5 days by visual inspection, semi-quantitatively evaluated by densitometric analysis, and shown as the relative hemolytic activity in the 33277 strain in relation to the A7436 strain (the latter set as 1.0). To better visualize and analyze hemolysis, bacterial colonies were removed. BM medium was used as a negative hemolysis control. (**E**) Gingipain activities of whole *P. gingivalis* cultures were measured using arginine-specific (Rgp) and lysine-specific (Kgp) substrates. Enzymatic activity was determined as an increase in absorbance at 405 nm per 1 minute per 1 µL of bacterial culture of optical density at 600 nm equal to 1. (**F**) Heme-binding ability by *P. gingivalis* cells. (**G**) Biofilm formation on the abiotic surface was examined using crystal violet staining and absorbance measurement at 570 nm. Insets in the graphs show a relative increase in heme binding (**F**) or biofilm formation (**G**) in iron- and heme-depleted conditions (DIP medium) in comparison to iron and heme-replete conditions (Hm medium). *P < 0.05, **P < 0.01, ***P < 0.001, ****P < 0.0001.

Phenotypic characterization of *P. gingivalis* growth in liquid culture media in iron- and heme-depleted or iron- and heme-replete conditions confirmed that the A7436 strain grew faster and its growth resulted in higher biomass production, as compared to the 33277 strain (Fig. 5A; Table S3). This result is not surprising because differences in the growth rate, including W83 and A7436 strains, have also been observed by others (72), suggesting that even strains belonging to the same group may phenotypically differ. When bacteria had been starved of iron and heme, subsequent growth in the presence of heme (DIP + Hm) or hemoglobin (DIP + Hb) was more efficient in the case of the A7436 strain as compared to the 33277 strain, the latter often being unable to recover and proliferate (Fig. 5B; Table S3). These results may indicate that the 33277 strain is less resistant to iron and heme starvation due to disturbed iron uptake and/or homeostasis. To explain this phenomenon, we determined intracellular iron content. The 33277 strain contained ~25% less intracellular iron than the A7436 strain (Fig. 5C). When *P. gingivalis* was grown on ABA plates, no significant differences in the formed pigment were observed (data not shown). The only difference was ~50% higher hemolysis caused by the 33277 strain in comparison to the A7436 strain (Fig. 5D). To find the bases for this phenotypic difference, we analyzed gingipain activity. Neither Rgp nor Kgp activity differed significantly when bacteria were grown either in iron- and heme-replete or in iron- and heme-depleted conditions (Fig. 5E), confirming that gingipain activity does not depend on iron and heme availability but rather on growth phase and bacterial density (73). The Rgp activity was similar in both tested strains; however, the 33277 strain exhibited two times higher Kgp activity than the A7436 strain in the two tested conditions (Fig. 5E). Since mainly Kgp is responsible for hemoglobin degradation, these results may explain the higher hemolytic activity of the 33277 strain.

Production of some *P. gingivalis* proteins, including outer membrane virulence factors (such as HmuY), is dependent on iron and heme availability (55). We observed that the 33277 strain, compared to the A7436 strain, bound more heme when cultured in iron- and heme-replete conditions (Fig. 5F). When both strains were cultured in iron- and heme-depleted conditions, although the general amount of bound heme was higher in 33277 than A7436 strain, the differences were not statistically significant (Fig. 5F).

In addition, we performed a comparative analysis of A7436 and 33277 strains in terms of their ability to form biofilm. Generally, biofilm formation by *P. gingivalis* is enhanced when bacteria are grown with reduced iron and heme content in the external environment. As expected (74), the more fimbriated 33277 strain formed larger biofilm structures, as compared to the A7436 strain (Fig. 5G). Moreover, both strains formed larger biofilm structures when bacteria had been starved of iron and heme; however, relative biofilm formation ability increased by ~2.25× for the A7436 strain and only ~1.45× for the 33277 strain (Fig. 5G). Although the results are not that profound, it is worth noting that we observed a higher increase in heme-binding ability caused by culturing the A7436 strain in iron- and heme-depleted conditions (~1.35× increase) as compared to iron- and heme-rich conditions, while for the 33277 strain, the increase was only ~1.2×. Moreover, these results can explain, at least in part, the differences in HmuY levels. Relative HmuY production in the DIP medium was ~35× and ~6× higher in A7436 and 33277 strains, respectively, in comparison to the Hm medium (Fig. 3C; Fig. S1). Altogether, this shows that iron and heme depletion has a greater impact on the phenotype presented by the A7436 strain than the 33277 strain. Therefore, our results suggest that not only the availability of iron and heme in the external environment but also the ability to store iron intracellularly can influence the *P. gingivalis* gene expression and phenotype.

### Hemoglobin influences the interaction of A7436 and 33277 strains with keratinocytes differently

**Fig 6:**
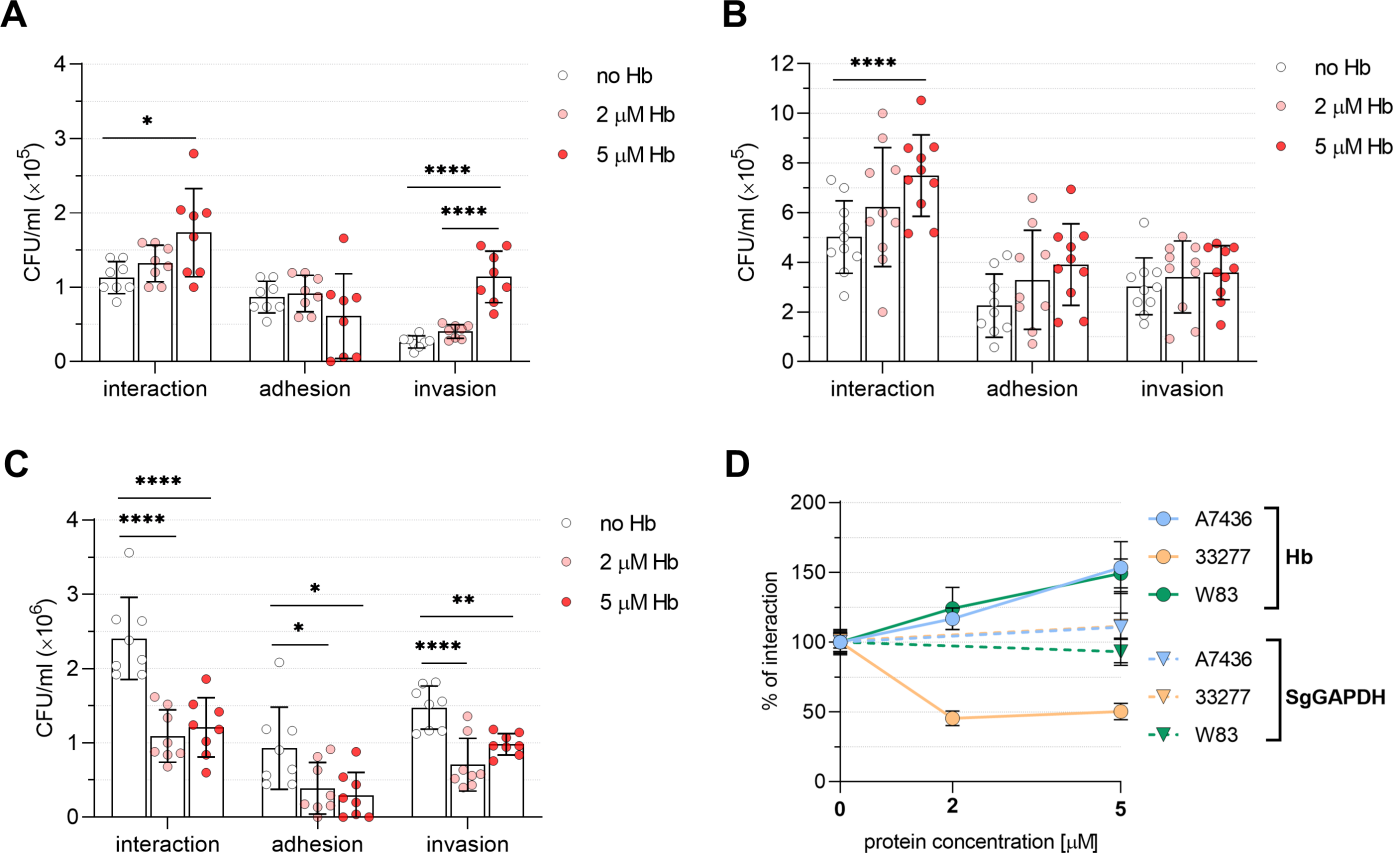
Influence of hemoglobin on interaction with host cells of *P. gingivalis* A7436 (**A**), W83 (**B**), and ATCC 33277 (**C**) strains. The ability to interact with, adhere to, and invade host cells was analyzed using a *P. gingivalis*-gingival keratinocytes co-culture model. The number of viable bacteria was shown as the number of colony-forming units (CFU) per mL of the culture medium. Adhesion—live bacteria attached to keratinocytes; invasion—live bacteria that invaded keratinocytes; interaction—the total number of live bacteria that adhered to and invaded keratinocytes. (**D**) Summary of the correlation between hemoglobin concentration and *P. gingivalis* interaction with human keratinocytes. The average interaction of *P. gingivalis* with keratinocytes in samples without hemoglobin was set as 100% for each tested strain. As a protein control, purified apo-SgGAPDH protein instead of hemoglobin was used. Results are shown as mean ± SD (**A through C**) or as mean ± SE (**D**). Hb—hemoglobin; SgGAPDH—glyceraldehyde-3-phosphate dehydrogenase from *Streptococcus gordonii*. *P < 0.05, **P < 0.01, ****P < 0.0001.

The availability of hemoglobin, as the main source of iron and PPIX, varies at different stages of periodontitis, being highly available in advanced stages of the disease (55, 56). Therefore, in this study, we not only compared the interaction of A7436 and 33277 strains with human gingival keratinocytes but also examined the influence of hemoglobin concentration on the adhesion to and invasion of keratinocytes. Also for this experiment, we used the reference W83 strain. The general interaction ability of all strains with keratinocytes was as follows: 33277 > W83 > A7436 (Fig. 6A through C). Increasing concentration of hemoglobin increased the interaction ability of more virulent A7436 and W83 strains. In the case of A7436, hemoglobin increased its invasion ability, and in the case of the W83 strain both adhesion and invasion abilities (Fig. 6A and B). The addition of 2 µM and 5 µM hemoglobin resulted in an increase in the growth of A7436 and W83 strains in the presence of keratinocytes by ~25% and ~50%, respectively (Fig. 6D). Conversely, increasing the concentration of hemoglobin decreased the growth, adhesion, and invasion abilities of the 33277 strain by ~50% (Fig. 6C and D). This finding was quite surprising in light of higher hemolytic and Kgp activities presented by the 33277 strain (Fig. 5D). As a control, we supplemented *P. gingivalis*-keratinocyte co-cultures with purified apo-SgGAPDH protein. However, we did not observe any significant changes in this case (Fig. 6D). Overall, our results showed that hemoglobin availability may influence the adhesion to and invasion of keratinocytes of some *P. gingivalis* strains in a concentration-dependent manner.

## DISCUSSION

*P. gingivalis* is one of the most important microbial agents involved in the initiation and progression of periodontitis (1, 14–17). The bacterium produces numerous virulence factors, enabling it to survive in different niches of the human body and infect host cells (55). However, it has been shown that *P. gingivalis* can also be present in healthy humans (e.g., 28, 30, 32, 42), which may suggest that not all *P. gingivalis* strains are pathogenic or their contribution to this process is of minor importance (75). Indeed, it has been shown that during evolution *P. gingivalis* strains may have diverged into groups varying in their genotypes and phenotypes, resulting in different virulence potentials (e.g., 28–30, 32–35, 40–43, 76, 77). The diversity of *P. gingivalis* strains results from their genetic variability caused mainly by the rearrangement of the bacterial chromosome. This seems to be possible because *P. gingivalis* encodes many transposon sequences that are responsible for this process and their number differs between strains (29). Moreover, recent studies showed that clustered regularly interspaced short palindromic repeat (CRISPR) and CRISPR-associated genes (Cas) (CRISPR-Cas) can influence virulence due to their engagement in transcription regulation or biofilm formation (44, 78–81).

In general, *P. gingivalis* strains are classified as virulent/more virulent (e.g., A7436, W83, W50) or as less virulent/avirulent (e.g., ATCC 33277, 381, HG66). The most studied, more virulent A7436 and W83 strains, belong to encapsulated strains, whereas the less virulent ATCC 33277 strain does not produce a capsule (22, 28, 33, 36–39, 82, 83). *P. gingivalis* produces major and minor fimbriae, the main components of which, the FimA and Mfa1 proteins, respectively, differ significantly in their structures (24, 30, 31, 39, 83–87). Moreover, different types of fimbriae are produced by *P. gingivalis* strains, which is often correlated with pathogenicity (87, 88). The bacterium also produces different LPS isoforms with significant structural variations of lipid A and O-antigen moieties that can affect its pro-inflammatory potential (89–91).

While the genomes of the A7436 and W83 strains are similar, significant differences between them and the 33277 genome were reported (44, 82, 83, 92, 93). For example, in comparison to the 33277 strain, both A7436 and W83 strains can survive much longer intracellularly after entering human endothelial cells *in vitro* (28). However, in contrast to the W83 strain, the A7436 strain traffics through different intracellular pathways and is not able to induce autophagy (26). Also increased colonization of rat tissues caused by the A7436 strain, as compared to the 33277 or W83 strain, has been reported (94, 95). Although W83 and A7436 genomes are quite similar, the strains exhibit some differences in the observed phenotypes and virulence potential (26).

Heme is a limiting factor for *P. gingivalis* growth and is essential for its survival. It is worth mentioning here that heme availability changes during the disease progress. Moreover, the source of heme depends on the niche occupied by the bacterium (55, 56). In addition, different heme concentrations affect various *P. gingivalis* strains differently, resulting in different tolerance to heme (61). To date, several studies have documented the influence of iron and heme availability on gene expression in *P. gingivalis* (e.g., 96, 97). Our additional analyses, including the A7436 strain, not previously studied in this aspect, showed that iron and heme may contribute differently to gene expression in *P. gingivalis* strains (Tables S1 and S2; Table 2). Some of the genes only partially overlapped (Fig. 2), which may indicate different mechanisms leading to the adaptation of different *P. gingivalis* strains due to the changing availability of iron and heme.

Heme uptake systems play a key role in the adaptation of *P. gingivalis* to the niche it occupies. Interestingly, the genetic variation in these systems is negligible between *P. gingivalis* strains, showing that they are crucial for the bacterium. Our phenotypic characterization of examined strains grown in iron- and heme-replete or in iron and heme-depleted conditions showed that expression of the Hmu system in both tested strains is highly dependent on iron and heme availability, whereas expression of other *P. gingivalis* putative heme uptake systems, such as Iht and Hus systems, is not iron- and heme-dependent (Fig. 3). Iron-dependent regulation in bacteria is typically mediated by a ferric uptake regulator (Fur). Although we have shown that a *P. gingivalis* Fur homolog (PgFur) does not function like the classic Fur protein, it regulates the production of HmuY protein (98, 99). Moreover, we observed that PgFur has a different effect on the regulation of virulence factors in 33277 and A7436 strains (100). It seems that the 33277 strain exhibits a lower ability to accumulate iron intracellularly (Fig. 5C), which may directly affect its susceptibility to starvation from this element, and the production of proteins whose regulation is dependent on this ion. One example is the HmuY protein, the production of which is directly related to the availability of iron, being produced in higher amounts after iron and heme starvation (55). We found that in iron- and heme-replete conditions production of HmuY is higher in the 33277 strain as compared to the A7436 strain (Fig. 3). Such an effect might be caused by the lower amount of intracellular iron in the 33277 strain, which stimulates the bacterial cell to produce proteins responsible for iron acquisition, including higher expression of the HmuY protein. This might result, at least in part, in the higher adsorption of heme on the surface of the 33277 strain grown in iron- and heme-rich conditions (Fig. 5F). We observed a similar effect for the IhtB protein, production of which is in general higher in the 33277 strain than in the A7436 strain (Fig. 3). However, in contrast to HmuY, IhtB production is not regulated by PgFur (98). An additional potential explanation of the differences observed between the examined strains in iron and heme homeostasis and their influence on the phenotype is the lack of the complete two-component regulatory system (HaeSR) in the 33277 strain, which is engaged in other strains in the regulation of expression of the *hmu* and *iht* operons, as well as in the regulation of expression of genes encoding gingipains (101).

The interaction of *P. gingivalis* with other bacteria and host cells is essential for an efficient virulence process, often dependent on the proteins that are components of the outer membrane, for example, fimbriae, hemagglutinins, and gingipains. In general, *P. gingivalis* tends to form higher biofilm structures under iron and heme starvation in comparison to conditions of iron and heme excess (74), a feature also confirmed in this study. Moreover, we also found that iron and heme starvation leads to a greater increase in biofilm formation by the A7436 strain in comparison to the 33277 strain. We think that differences in iron and heme homeostasis play an additional role in different *P. gingivalis* strains’ phenotypes. Moreover, the ability of the A7436 strain to accumulate more iron intracellularly may provide it with a greater capacity to adapt to changing environmental conditions, including stress conditions, such as iron and heme deficiency. Consequently, it could lead to quantitative and qualitative differences in the composition of the outer membrane of particular strains, thus influencing the ability to interact with host cells differently. In the advanced stage of periodontitis, the main source of heme for *P. gingivalis* is hemoglobin, which is present in large amounts after lysis of erythrocytes. Therefore, its concentration may affect the expression of heme-dependent genes in *P. gingivalis*. The presence of many proteins, including gingipains, hemagglutinins, HmuY, and HmuR, affects the binding of heme and hemoglobin on the surface of the *P. gingivalis* cell. Our data showed that the interaction with host cells of the strains can vary depending on the amount of free hemoglobin in the external environment. We noted an increase in the adhesion to and invasion of keratinocytes of A7436 and W83 strains with an increasing hemoglobin concentration but the opposite effect for the 33277 strain (Fig. 6). Another explanation of this effect could be the lower resistance of the 33277 strain, as compared to the A7436 strain, to oxidative stress (102). The addition of hemoglobin under aerobic conditions used in our study may generate an increase in reactive oxygen species, which might affect the survival of bacteria. This may prove the better adaptation ability of the A7436 strain to the changing conditions of the external environment, as compared to the 33277 strain.

We found that the 33277 strain produced a higher amount of HmuY in iron- and heme-replete conditions, and generally produced higher amounts of IhtB, as well as Kgp. The influence of the latter protein, besides higher Kgp enzymatic activity, could also be responsible for the increased hemagglutinating activity. Therefore, a higher amount of heme- and hemoglobin-binding proteins on the surface of the 33277 strain may result in higher heme and hemoglobin binding, thus preventing adhesion to host cells and subsequent entry into keratinocytes. Moreover, besides proteinaceous components of the outer membrane, differences in LPS structures, mainly in lipid A, might be responsible for strains’ variability (89, 101–104). It was reported that differences in both fimbriae and LPS between *P. gingivalis* strains can result in different hydrophobicity of strains, which may cause various recognition of molecules produced by cohabitating bacteria, different co-aggregation abilities, and different capacities for biofilm formation, and possibly differences in heme and hemoglobin binding by outer membrane molecules (103–108). Gingipains are modified by the APS (58, 90, 91, 109, 110). Therefore, we assumed that differences in molecular weight observed for pro-RgpB (Fig. 3 and 4C) could be due to the changes in LPS structures. Both A7436 and 33277 strains differ in glycosylation (100), which could, at least in part, explain the effect observed in the examined strains (Fig. 3C, D, and 4C,D).

In conclusion, accumulating evidence demonstrates that *P. gingivalis* strains exhibit different phenotypes *in vitro*, different virulence potential in animal models, and different associations with human diseases. We also observed that the less virulent 33277 strain and more virulent A7436 strain exhibit different phenotypes in regard to iron and heme acquisition, which may be caused by their genetic divergence. We think that the ability to accumulate iron intracellularly, and resistance to iron and heme starvation being different in the examined *P. gingivalis* strains, may exert a significant impact on their observed phenotypes, influencing the production of virulence factors and the virulence potential of *P. gingivalis* strains. We believe that the results presented in this study shed new light on differences between *P. gingivalis* strains and their virulence potential to cause dysbiosis in the oral cavity.

## MATERIALS AND METHODS

### Bacterial strains and growth conditions

*Porphyromonas gingivalis* wild-type A7436, ATCC 33277 (33277) and W83 strains were maintained on Schaedler blood agar (ABA) plates (Biomaxima, Lublin, Poland) in anaerobic conditions (80% N_2_, 10% H_2_, and 10% CO_2_) (Whitley A35 anaerobic workstation; Bingley, UK) at 37°C for 3–5 days. Then, bacteria were inoculated into liquid basal medium (BM) prepared from 3% trypticase soy broth (Becton Dickinson, Sparks, MD, USA) and 0.5% yeast extract (Biomaxima), supplemented with 3.6 mM L-cysteine hydrochloride (Carl Roth, Karlsruhe, Germany), 0.5 mg/L menadione (Sigma-Aldrich, St. Louis, MO, USA), and 7.7 µM hemin chloride (Fluka, Munich, Germany) (Hm medium). To examine the influence of iron and heme on gene expression, bacteria were grown in two types of liquid media. To imitate bleeding occurring in individuals with an advanced stage of periodontitis (characterized by high concentrations of iron and heme in the oral cavity), bacteria were grown in Hm medium. To imitate the conditions of a healthy oral cavity (characterized by trace amounts of iron and heme), 160 µM of the iron chelator 2,2-dipyridyl (Sigma-Aldrich) (DIP medium) was used instead of hemin chloride. To monitor *P. gingivalis* growth, bacteria were inoculated into Hm or DIP medium with a starting optical density at 600 nm (OD_600_) equal to 0.2. *P. gingivalis* growth was monitored using a Stratus plate reader (Cerillo, Charlottesville, VA, USA) as described previously (56).

### Biofilm formation

To compare the biofilm-forming capacity of *P. gingivalis* 33277 and A7436 strains, a sterile 96-well plate (Corning, New York, US) was inoculated with 200 µL of bacterial cultures in Hm or DIP medium per well at starting OD_600_ equal to 0.2. Bacteria were cultured at 37°C under anaerobic conditions for 24 hours. Then, wells were washed three times with 20 mM sodium phosphate buffer, pH 7.4, containing 140 mM NaCl (phosphate-buffered saline, PBS) to remove unattached bacteria. The biofilm was stained with 1% crystal violet (Carl Roth, Karlsruhe, Germany) for 10 minutes, then washed five times with PBS and de-stained with 99.8% ethanol. Absorbance at 570 nm (A_570_) was measured using a GloMax Discover plate reader (Promega, Madison, WI, USA). The obtained values were normalized to the OD_600_ of the liquid bacterial cultures equal to 1.

### Microarray analysis of gene expression

*P. gingivalis* A7436 and 33277 strains were grown in three biological replicates in Hm and DIP media for 20 hours as described above. The microarray analysis was performed in IMGM laboratories (Martinsried, Germany) as described previously (99). Briefly, the online tool eArray (http://earray.chem.agilent.com/; Agilent Technologies, Santa Clara, CA, USA) was used to design an Agilent Custom *Porphyromonas gingivalis* A7436 Gene Expression Microarray (8 × 15K format). Probes were prepared based on *P. gingivalis* transcriptome information derived from the NCBI reference sequence NZ_CP011995.1. Total RNA isolation, RNA quantity, and quality were determined as described previously (99). For internal labeling control, the total RNA was spiked with *in vitro* synthesized polyadenylated transcripts (One-Color RNA Spike-In Mix; Agilent Technologies). Subsequently, samples were reverse transcribed into cDNA and then converted into cyanine-3-labeled complementary RNA (cRNA) with Low Input Quick-Amp Labeling Kit One-Color (Agilent Technologies). For microarray hybridization, a Gene Expression Hybridization Kit (Agilent Technologies) was used. Labeled cRNA was hybridized for 17 hours at 65°C on Agilent Custom GE 8 × 15K Microarrays, washed according to the manufacturer’s protocol, and dried with acetonitrile (Sigma-Aldrich). The fluorescence of samples was detected with Scan Control A.8.4.1 software (Agilent Technologies) on the Agilent DNA Microarray Scanner (Agilent Technologies) and extracted from the images using Feature Extraction 10.7.3.1 software (Agilent Technologies). For data analysis, Feature Extraction 10.7.3.1 (Agilent Technologies), GeneSpring GX 13.1.1 (Agilent Technologies), and Excel 2010 (Microsoft, Redmond, WA, USA) were used. For statistical analysis, Welch’s approximate *t*-test was used. Differences in gene expression are shown as fold change values (FC). The average was calculated from the normalized signal values and they were transformed from the log_2_ to the linear scale. Increases and decreases in gene expression are shown as positive and negative numbers, respectively. The fold change in gene expression was considered significant for FC ≥ 2 or FC ≤ −2 and *P*-value ≤ 0.05.

### Gene expression analysis examined using reverse transcriptase-quantitative polymerase chain reaction

*P. gingivalis* strains were maintained for 10 or 24 hours in Hm and DIP media. Total RNA was isolated with the Total RNA Mini Kit (A&A Biotechnology, Gdańsk, Poland), followed by the Clean-up RNA concentrator Kit (A&A Biotechnology). cDNA was obtained by reverse transcription using a LunaScript RT SuperMix Kit (New England Biolabs, Ipswich, MA, USA). For PCR, the SensiFAST SYBR no-ROX Kit (Bioline, London, UK) and LightCycler 96 (Roche, Basel, Switzerland) were used. The PCR program consisted of initial denaturation at 95°C for 120 seconds, 40 cycles of denaturation at 95°C for 5 seconds, primer annealing at 60°C for 10 seconds, and extension at 72°C for 15 seconds. For PCR product quality, the melting curves were generated and analyzed. All samples were examined in triplicate. Fold change in gene expression was calculated with LightCycler 96 software (Roche). All primers used in this study are listed in Table S4, including the *P. gingivalis 16S rRNA* gene used as the reference gene.

### IhtB protein purification and anti-IhtB antibody production

To generate anti-IhtB antibodies, the recombinant IhtB protein (GenBank accession number AKV64486), lacking the predicted signal peptide (MKKLILATLGLMAIAMLSCS), was purified. Briefly, the *ihtB* gene was PCR amplified using primers listed in Table S4. The PCR product was cloned into XcmI and BamHI restriction sites of a pTriEx-4 plasmid (Sigma-Aldrich) using NEBuilder HiFi DNA Assembly (New England Biolabs), enabling IhtB protein production with the 6×His tag fused to the N-terminus with the possibility to be removed with Xa factor. IhtB protein was overexpressed using *Escherichia coli* BL21-CodonPlus (DE3)-RIL strain (Agilent Technologies, Santa Clara, CA, USA) and purified from the soluble *E. coli* cell lysate using TALON Superflow resin according to the manufacturer’s protocol (Sigma-Aldrich). Subsequently, the 6×His-tag was cut off using the Xa factor according to the manufacturer’s protocol (New England Biolabs) and removed by washing and protein concentration using Amicon Ultra-4 Centrifugal Ultracel-10KDa filter units (Millipore).

Anti-IhtB antibodies were produced in rabbits using purified IhtB protein as an antigen, according to the company’s protocol (ProteoGenix, Schiltigheim, France).

### Sodium dodecyl sulfate-polyacrylamide gel electrophoresis, Western blotting, and lectin assay

Sodium dodecyl sulfate-polyacrylamide gel electrophoresis (SDS-PAGE) samples were prepared using bacteria grown in liquid and on solid media as reported previously (56). Bacterial lysates were separated on 12% polyacrylamide gels using SDS-PAGE, transferred onto nitrocellulose membranes (Millipore, Billerica, MA, USA), and subsequently probed with rabbit antibodies raised against HmuY (1:10,000; GenScript USA Inc.), IhtB (1:10,000; ProteoGenix), or the RgpB catalytic domain (1 µg/mL; Cusabio; Houston, TX, USA). Then, goat anti-rabbit IgG antibodies conjugated with horseradish peroxidase (1:20,000; Sigma-Aldrich) were used.

To analyze potential differences in glycosylation of macromolecules, a lectin assay was performed as reported previously (100). Briefly, whole cell lysates were separated by SDS-PAGE, transferred onto nitrocellulose membranes, and probed with JACALIN lectin (1:5,000) specific to galactose linked with β−1,3 linkage to *N*-acetylgalactosamine and mono- or di-sialylated form of this structure (Vector Laboratories, Newark, CA, USA). Lectin binding was detected using avidin D conjugated with horseradish peroxidase (1:10,000; Vector Laboratories). Equal loading of proteins was visualized on nitrocellulose membranes by staining with Ponceau S (data not shown).

Western blotting and lectin assay signals were visualized using chemiluminescence staining (Perkin Elmer, Waltham, MA, USA) and a ChemiDoc Imaging System (Bio-Rad Laboratories, Hercules, CA, USA). Densitometric analysis (relative quantification) was performed using Image Lab 6.0.1 software (Bio-Rad).

### Determination of intracellular iron content

Intracellular iron concentration was determined using the ferrozine-based method as described previously (56). Briefly, aliquots of overnight bacterial cultures (BM + Hm; 20 mL) were centrifuged and washed with PBS, and bacterial suspensions (4 mL) at OD_600_ equal to 2.0 were sonicated. Samples were then treated according to the procedure reported by Ceriotti and Ceriotti (111). The absorbance of the supernatants was measured at 562 nm. The iron content was determined from a standard curve prepared similarly to bacterial samples using FeCl_3_ (Sigma Aldrich).

### Heme binding by the whole *P. gingivalis* cells

The ability of heme binding to whole *P. gingivalis* cells was performed as described previously (56). Briefly, aliquots of overnight bacterial cultures (BM + Hm or BM + DIP; 20 mL) were centrifuged and washed with PBS, and bacterial suspensions (0.8 mL) at OD_600_ equal to 1.25 were mixed with 0.2 mL of hemin chloride in PBS (50 µg/mL). After incubation for 1 h at 37°C, the samples were centrifuged and the absorbance of the supernatant was measured at 385 nm. Control samples contained all components except bacteria. The amount of heme bound to bacterial cells was determined from the difference in absorbance between the control and bacterial samples.

### Determination of gingipain activity

Gingipain activity was measured as reported by others (112) with minor modifications. Briefly, to 150 µL of the reaction buffer (20 mM TRIS pH 7.5, supplemented with 150 mM NaCl, 0.05% Tween 20, 5 mM CaCl_2_, and 10 mM L-cysteine hydrochloride, neutralized with NaOH), 10 µL of *P. gingivalis* cultures was added and samples were incubated for 15 minutes at 37°C. The reaction was started by addition of 50 µL of reaction buffer, supplemented with 2 mM N-(p-tosyl)-Gly-Pro-Lys 4-nitroanilide acetate salt (Sigma-Aldrich) for determination of the Kgp activity or 2 mM Nα-benzoyl-DL-arginine p-nitroanilide hydrochloride (BApNA; Sigma-Aldrich) for determination of the Rgp activity. The samples were incubated for 2 hours at 37°C and the reaction was monitored by measuring the increase of absorbance at 405 nm (A_405_) over time using a GloMax Discover plate reader (Promega). Rgp or Kgp activities are shown as an increase in absorbance at 405 nm (A_405_) per minute per 1 µL of the bacterial culture at an optical density at 600 nm (OD_600_) equal to 1 (mOD/min/μL).

### Hemolytic activity

Aliquots of 25  µL of overnight liquid bacterial cultures (BM + Hm) at OD_600_ equal to 2 were applied on ABA plates in spots of ~1 cm diameter and cultured for 5  days. Bacteria were removed from plates and relative hemolytic activity was determined with the ChemiDoc Imaging System (Bio-Rad) using densitometric analysis (relative quantification) with the Image Lab 6.0.1 software (Bio-Rad).

### Interaction with host cells

Immortalized human gingival keratinocytes (Gie-No3B11; ABM, Richmond, British Columbia, CA, USA) were used as a host cell model. Keratinocytes and bacteria were grown as described previously (56, 113). Keratinocytes were seeded (1.0 × 10^4^ cells per well) in 24-well plates (Corning, New York, US) and grown for 24 hours in TM-040 medium (ABM), supplemented with 2% heat-inactivated fetal bovine serum (FBS, Cytogen, Zgierz, Poland), 100 U/mL penicillin (Sigma-Aldrich) and 100 µg/mL streptomycin (Cytogen) at 37°C in an atmosphere of 5% CO_2_.

To determine the influence of hemoglobin on *P. gingivalis* interaction with keratinocytes, human hemoglobin (Sigma-Aldrich) was used. As a control, another heme-binding protein, namely *Streptococcus gordonii* glyceraldehyde-3-phosphate dehydrogenase (SgGAPDH), was used in its apo form. SgGAPDH was overexpressed and purified as described previously (114). To remove endotoxins, Detoxi-Gel Endotoxin Removing Columns were used according to the manufacturer’s protocol (Thermo Scientific).

*P. gingivalis* was grown to the early stationary phase in Hm medium, centrifuged (4,000 × *g*, 20 minutes, 4°C), and washed twice with PBS. Keratinocytes were washed three times with PBS and a fresh DMEM medium (Sigma-Aldrich) or DMEM medium supplemented with a sterile solution of human hemoglobin or SgGAPDH at the final concentration of 2 µM or 5 µM. The cells were treated with *P. gingivalis* with the multiplicity of infection (MOI) of 100 and incubated for 4 hours at 37°C in an atmosphere of 5% CO_2_. Then, the wells were washed three times with PBS. To determine the bacteria present inside the cells and attached to them, a fresh DMEM medium was added. To kill the external bacteria and to determine only live bacteria inside the cells, DMEM medium supplemented with 300 µg/mL gentamicin (Sigma-Aldrich) and 200 µg/mL metronidazole (Sigma-Aldrich) was added. After 1 hour, the wells were washed three times with PBS, and lysed with sterile distilled water. The cell lysates were used to prepare serial dilutions, which were plated on ABA plates. The plates were incubated for 7 days at 37°C in anaerobic conditions to determine colony-forming units (CFU).

### Bioinformatics analyses

The sequences used for comparisons were retrieved from the GenBank database. To analyze the sequences’ homology, Clustal Omega (115) was used. Genome IDs and operon *hmu* loci IDs for *P. gingivalis* are listed in Table 1, and for *Porphyromonas gulae* GenBank sequence ID (KB899153.1) and *hmu* operon locus ID (F452_RS0103495 – F452_RS0103470) were used for DSM 15663 strain. To analyze RgpB protein, the amino acid sequences were obtained for A7436 (ID: AKV64625), W83 (ID: AAQ65700), and ATCC 33277 (ID: AUR49078) strains and compared with sequences’ alignment using Clustal Omega and Jalview (116). To visualize the phylogenetic tree, The EvolView server was used (117). *P. gingivalis* ATCC 33277, A7436, and W83 genomes were visualized using Proksee (https://proksee.ca/).

### Statistical analyses

Except for microarray analysis, all statistical analyses were performed with GraphPad software (GraphPad Prism 8.0 Inc., San Diego, CA, USA) using the unpaired Student’s *t*-test or one-way analysis of variance (ANOVA) test with post hoc Tukey’s test. All experiments were conducted at least three times with at least two biological replicates and collected data are shown as mean ± standard deviation (mean ± SD) or as mean ± standard error (mean ± SE).

## Data Availability

Microarray data are available at Zenodo (accession no: 10118417)

## SUPPLEMENTAL MATERIAL

Tables S1-S4 spectrum.02865-23-s0001.pdf Supplementary data.

Fig. S1 and S2 spectrum.02865-23-s0002.pdf Supplementary data.

ASM does not own the copyrights to Supplemental Material that may be linked to, or accessed through, an article. The authors have granted ASM a non-exclusive, world-wide license to publish the Supplemental Material files. Please contact the corresponding author directly for reuse.
