# Supplementary material for: Comparative analysis of Porphyromonas gingivalis A7436 and ATCC 33277 strains reveals differences in the expression of heme acquisition systems
Source: Microbiol Spectr. 2024 Jan 30;12(3):e02865-23. doi: 10.1128/spectrum.02865-23 (PMC10913741; doi:10.1128/spectrum.02865-23)
Supplement: Tables S1-S4 — Supplementary data. [file spectrum.02865-23-s0001.pdf]

SUPPLEMENTAL MATERIAL

Table S1 Analysis of gene expression in *P. gingivalis* grown for 20 hours in iron and heme-depleted conditions (DIP medium) in comparison to iron and heme-replete conditions (Hm medium) examined using microarray analysis – up-regulated genes.

**Locus ID**			**Gene name**	**A7436**	**33277**	**Gene description**	**Category**
**W83**	**A7436**	**33277**		**Fold change (FC)**			
PG0063	PGA7_RS00290	PGN_2012		23.71±3.92	6.10±0.15	TolC family protein	c
PG0064	PGA7_RS00295	PGN_2013		15.39±4.20	6.06±2.36	multidrug transporter AcrB	c
PG0090	PGA7_RS00405	PGN_2037	*dps*	3.31±0.10	5.22±0.13	DNA protection during starvation protein	r
PG0421	PGA7_RS07135	PGN_1547		3.10±0.28	4.06±0.71	DUF2807 domain-containing protein	c
PG0485	PGA7_RS06850	PGN_1485	*yajC*	66.83±38.46	11.90±5.37	T9SS C-terminal target domain-containing protein	p
PG0618	PGA7_RS06280	PGN_0660	*ahpC*	8.78±0.82	11.85±1.10	alkyl hydroperoxide reductase subunit C	c
PG0619	PGA7_RS06275	PGN_0661	*ahpF*	9.66±0.63	15.60±1.66	alkyl hydroperoxide reductase subunit F	c
PG0686	PGA7_RS05980	PGN_0722		4.55±0.49	2.97±0.95	hypothetical protein	h
PG0707	PGA7_RS05895	PGN_0741		8.01±3.13	2.74±0.39	TonB-dependent receptor	t
PG0928	PGA7_RS04910	PGN_1019	*porX*	2.63±0.21	2.79±0.61	PglZ domain-containing protein	r
PG1019	PGA7_RS04485	PGN_1336		133.18±26.37	73.86±28.71	DUF4876 domain-containing protein	h
PG1020	PGA7_RS04480	PGN_1335		116.64±18.57	26.76±5.33	TonB-dependent receptor	r
PG1022	PGA7_RS04475	PGN_1334		81.73±6.88	18.81±3.54	hypothetical protein	h
PG1043	PGA7_RS04380	PGN_1309	*feoB*	2.23±0.09	3.07±0.42	ferrous iron/manganese transporter B	t
PG1044	PGA7_RS04375	PGN_1308	*feoA*	3.95±0.35	8.00±1.32	DtxR family transcriptional regulator with two FeoA domains	r
PG1124	PGA7_RS04040	PGN_1221		2.15±0.03	2.75±0.07	cobalamin adenosyltransferase	b
PG1175	PGA7_RS03815	PGN_0950		8.35±1.44	37.54±3.88	ABC transporter ATP-binding protein	t
PG1176	PGA7_RS03810	PGN_0949		8.93±0.94	43.57±6.75	ABC transporter ATP-binding protein	t
PG1178	PGA7_RS03805	PGN_0948		9.07±1.17	130.57±56.12	hypothetical protein	h
PG1179	PGA7_RS03800	PGN_0947		9.71±1.04	111.93±33.68	outer membrane lipoprotein-sorting protein	l
PG1180	PGA7_RS03795	PGN_0946		12.83±2.02	85.13±26.49	membrane protein	t
PG1181	PGA7_RS03790	PGN_0945		30.19±3.44	131.74±32.94	TetR family transcriptional regulator	r
PG1236	PGA7_RS03595	PGN_1372		4.37±0.89	8.18±3.51	cation-binding protein	h
PG1237	PGA7_RS03590	PGN_1373		6.26±0.89	12.63±4.52	LuxR family transcriptional regulator	r
PG1294	PGA7_RS03080	PGN_1085	*feoB*	10.57±4.25	2.47±0.08	ferrous iron/manganese transporter B	t
PG1374	PGA7_RS02720	PGN_0852		9.30±7.17	3.55±0.46	T9SS C-terminal target domain-containing protein	o
PG1408	PGA7_RS02580	PGN_0886		4.08±0.75	2.78±0.18	cobalt transporter	c
PG1539	PGA7_RS02105	PGN_0570	*truB*	2.45±0.38	2.09±0.02	tRNA pseudouridine synthase B	p
PG1551	PGA7_RS02055	PGN_0558	*hmuY*	196.09±26.54	17.31±3.03	heme-binding protein HmuY	v
PG1552	PGA7_RS02050	PGN_0557	*hmuR*	49.07±7.70	7.29±2.38	TonB-dependent receptor	t
PG1553	PGA7_RS02045	PGN_0556	*hmuS*	71.83±21.01	4.94±0.76	cobaltochelatase	b
PG1555	PGA7_RS02035	PGN_0554	*hmuU*	75.98±6.57	7.32±0.91	MotA/TolQ/ExbB proton channel family protein	t
PG1556	PGA7_RS02030	PGN_0553	*hmuV*	81.83±7.73	5.56±0.17	hypothetical protein	t

PG1642	PGA7_RS01670	PGN_0493		4.64±0.35	5.04±0.11	cation transporter	o
PG1827	PGA7_RS08145	PGN_1740	*sigH*	2.37±0.25	3.47±0.46	RNA polymerase sigma factor	r
PG1857	PGA7_RS08255	PGN_1790		93.66±10.07	37.50±1.63	hypothetical protein	h
PG1858	PGA7_RS08260	PGN_1791		99.01±27.63	27.82±5.63	flavodoxin	e
PG1868	PGA7_RS08290	PGN_1797		5.94±0.21	28.03±2.14	membrane protein	o
PG1870	PGA7_RS08295	PGN_1798		14.86±1.85	85.10±17.44	methyltransferase UbiE	b
	PGA7_RS06905	PGN_1496		2.56±0.09	2.14±0.20	hypothetical protein	h
PG0003	PGA7_RS00015	PGN_0003		3.71±0.50	ND	acyltransferase family protein	h
PG0005	PGA7_RS00025	PGN_0005		3.23±0.82	ND	metallophosphoesterase	h
PG0065	PGA7_RS00300	PGN_2014		28.13±2.75	ND	efflux RND transporter periplasmic adaptor subunit	c
PG0144	PGA7_RS00665	PGN_0257		2.04±0.03	ND	agmatine deiminase family protein	i
PG0167	PGA7_RS00770	PGN_0279	*rplY*	2.24±0.04	ND	50S ribosomal protein L25/general stress protein Ctc	p
PG0209	PGA7_RS00930	PGN_0314		10.55±3.20	ND	formate/nitrite transporter family protein	t
PG0219	PGA7_RS00985			2.36±0.20	ND	hypothetical protein	h
PG0228	PGA7_RS01020	PGN_0331		2.53±0.39	ND	arginine deiminase family protein/amidinotransferase	h
PG0350	PGA7_RS07445	PGN_1611	*inlJ*	3.38±0.24	ND	leucine-rich repeat domain-containing protein	v
PG0425	PGA7_RS07110	PGN_1542		3.00±0.95	ND	esterase family protein	h
PG0497	PGA7_RS06810	PGN_1475	*mtn*	2.77±0.04	ND	5'-methylthioadenosine/adenosylhomocysteine nucleosidase	i
PG0506	PGA7_RS06770	PGN_1466	*rgpB*	2.53±0.28	ND	Arg-gingipain RgpB/Peptidase family C25	v
PG0523	PGA7_RS06690	PGN_1449		2.20±0.13	ND	IMP dehydrogenase/GMP reductase domain	n
PG0531	PGA7_RS06660	PGN_1441	*nadE*	2.38±0.22	ND	NAD(+) synthase/carbon-nitrogen hydrolase	b
PG0598	PGA7_RS06350	PGN_0642		2.78±0.70	ND	LptF/LptG family permease	t
PG0615	PGA7_RS06290	PGN_0658	*typA*	2.78±0.20	ND	translational GTPase TypA	r
PG0616	PGA7_RS06285	PGN_0659	*hbp35*	2.46±0.13	ND	HBP35, 35 kDa heme-binding protein, thioredoxin	t
PG0652	PGA7_RS06125	PGN_0691		2.36±0.12	ND	Dabb family protein/stress-responsive A/beta barrel domain	h
PG0664	PGA7_RS06085	PGN_0700		2.12±0.12	ND	Gfo/Idh/MocA family oxidoreductase	h
PG0665	PGA7_RS06080	PGN_0701		2.98±1.33	ND	beta-galactosidase	e
PG0676	PGA7_RS06030	PGN_0711		2.64±0.09	ND	SDR family oxidoreductase	a
PG0687	PGA7_RS05975	PGN_0723		4.69±2.41	ND	aldehyde dehydrogenase family protein	e
PG0715	PGA7_RS05855	PGN_0751	*corA*	3.22±0.55	ND	magnesium transporter CorA family protein	t
PG0752	PGA7_RS05695	PGN_0779	*upp*	2.52±0.24	ND	uracil phosphoribosyltransferase	n
PG0783	PGA7_RS05550	PGN_0807		2.16±0.11	ND	TatD family hydrolase/TatD-related DNase	h
PG0926	PGA7_RS04920	PGN_1021		2.20±0.11	ND	immunity 17 family protein	h
PG0975	PGA7_RS04680	PGN_0977		2.18±0.04	ND	PhoH family protein	r
PG1039	PGA7_RS04400	PGN_1313		3.44±0.77	ND	phosphoethanolamine transferase/sulfatase	l
PG1114	PGA7_RS04085	PGN_1204		3.10±0.03	ND	aspartate 1-decarboxylase	b
PG1132	PGA7_RS04000	PGN_1229		2.14±0.13	ND	valine-tRNA ligase	p
PG1133	PGA7_RS11715			2.41±0.21	ND	hypothetical protein	h
PG1137	PGA7_RS03970	PGN_1235	*porS*	3.26±0.51	ND	oligosaccharide flippase family protein	t
PG1174	PGA7_RS03820	PGN_1271		12.45±1.26	ND	PaaI family thioesterase	h
PG1205	PGA7_RS03720			3.70±1.06	ND	HU family DNA-binding protein	d

PG1235	PGA7_RS03600	PGN_1370		2.77±0.17	ND	NAD-dependent epimerase/dehydratase family protein	a
	PGA7_RS03075	PGN_1086		9.79±3.19	ND	FeoB-associated Cys-rich membrane protein	o
PG1296	PGA7_RS03070	PGN_1087		11.25±1.18	ND	PH domain-containing protein	h
PG1302	PGA7_RS03050	PGN_1091		2.13±0.10	ND	hypothetical protein	h
PG1315	PGA7_RS02985	PGN_1105	*slyD*	2.91±0.14	ND	peptidylprolyl isomerase	p
PG1331	PGA7_RS02920	PGN_1120		2.07±0.06	ND	Re/Si-specific NAD(P)(+) transhydrogenase subunit alpha	b
PG1335	PGA7_RS02895	PGN_1125		2.21±0.07	ND	NfeD family protein	h
PG1411	PGA7_RS02570	PGN_0889		2.06±0.06	ND	putative transporter/predicted permease membrane region	t
PG1465	PGA7_RS02440			25.49±6.51	ND	hypothetical protein	h
PG1466	PGA7_RS02435			27.7±8.11	ND	isoprenylcysteine carboxylmethyltransferase family protein	o
PG1467	PGA7_RS02430			27.06±7.66	ND	class I SAM-dependent methyltransferase	b
PG1519	PGA7_RS02195			2.61±0.22	ND	DUF4209 domain-containing protein	h
PG1536	PGA7_RS02120	PGN_0573		4.22±0.11	ND	permease-like cell division protein FtsX	c
PG1537	PGA7_RS02115	PGN_0572		2.97±0.18	ND	DUF3098 domain-containing protein	h
PG1538	PGA7_RS02110	PGN_0571	*uppP*	2.34±0.18	ND	undecaprenyl-diphosphate phosphatase	l
PG1554	PGA7_RS02040	PGN_0555	*hmuT*	57.1±11.29	ND	hypothetical protein	t
PG1616	PGA7_RS01770	PGN_0496		3.35±0.14	ND	fumarate reductase cytochrome b subunit	e
PG1662	PGA7_RS01595	PGN_0449		2.68±0.16	ND	hypothetical protein	h
PG1676	PGA7_RS01515	PGN_0434	*pckA*	2.49±0.10	ND	phosphoenolpyruvate carboxykinase (ATP)	e
PG1696	PGA7_RS01430	PGN_0416		2.36±0.14	ND	hypothetical protein	h
PG1758	PGA7_RS07825	PGN_1698	*rpsO*	3.29±0.11	ND	30S ribosomal protein S15	p
PG1788	PGA7_RS07980	PGN_1777		2.17±0.13	ND	aminopeptidase/Peptidase C1-like family	p
PG1854	PGA7_RS08240	PGN_1787		2.29±0.18	ND	5-formyltetrahydrofolate cyclo-ligase	i
PG1855	PGA7_RS08245	PGN_1788		2.74±0.77	ND	S41 family peptidase	p
PG1856	PGA7_RS08250	PGN_1789		2.92±0.79	ND	dCMP deaminase family protein	n
PG1890	PGA7_RS08375	PGN_1817		2.32±0.07	ND	hypothetical protein	h
PG1891	PGA7_RS08380			2.09±0.04	ND	hypothetical protein	h
PG1894	PGA7_RS08415			2.97±0.23	ND	hypothetical protein	h
PG1978	PGA7_RS08830			2.50±0.34	ND	hypothetical protein	h
PG1998	PGA7_RS08915			2.31±0.15	ND	polyprenyl synthetase family protein	c
PG2026	PGA7_RS09045	PGN_1973		2.31±0.07	ND	histidine phosphatase family protein	b
PG2027	PGA7_RS09050	PGN_1974		2.26±0.11	ND	hypothetical protein	h
PG2028	PGA7_RS09055	PGN_1975	*ybaK*	2.43±0.04	ND	aminoacyl-tRNA deacylase	p
PG2029	PGA7_RS09060	PGN_1976		2.14±0.08	ND	zinc-dependent metalloprotease/DUF4953	p
PG2085	PGA7_RS09340	PGN_0137	*trpS*	2.37±0.11	ND	tryptophan-tRNA ligase	p
PG2112	PGA7_RS09460	PGN_0162		2.26±0.10	ND	hypothetical protein	h
PG2120	PGA7_RS09485	PGN_0169		2.31±0.23	ND	beta-lactamase superfamily domain	p
PG2139	PGA7_RS09590	PGN_0187		2.86±0.33	ND	DUF177 domain-containing protein	h
PG2141	PGA7_RS09595	PGN_0189	*fabH*	2.36±0.13	ND	3-oxoacyl-ACP synthase III	f
PG2192	PGA7_RS09830			3.28±0.67	ND	peptidoglycan DD-metalloendopeptidase family protein	l
PG2200	PGA7_RS09865	PGN_2067		3.17±0.41	ND	tetratricopeptide repeat protein	t

PG2201	PGA7_RS09870	PGN_2068	*def*	2.69±0.24	ND	peptide deformylase	h
PG2004	PGA7_RS08945	PGN_1949		2.61±0.52	ND	putative sulfate exporter family transporter	t
PG0213	PGA7_RS00950	PGN_0318	*cobJ*	ND	3.39±0.42	precorrin-3B C(17)-methyltransferase	b
PG0275	PGA7_RS01205	PGN_0373		ND	2.17±0.12	thiol reductase thioredoxin	p
PG0433	PGA7_RS07075	PGN_1527		ND	2.54±0.09	S-adenosylmethionine-dependent methyltransferase	p
PG0536	PGA7_RS06615	PGN_1435		ND	3.18±0.07	hypothetical protein	h
PG0573	PGA7_RS06470	PGN_0620	*rsmH*	ND	3.20±0.04	ribosomal RNA small subunit methyltransferase H	p
PG0596	PGA7_RS06360	PGN_0640	*rpsR*	ND	2.04±0.03	30S ribosomal protein S18	p
PG0645	PGA7_RS06165	PGN_0684		ND	2.88±0.04	adenosylcobinamide amidohydrolase	b
PG0914	PGA7_RS04975	PGN_1032		ND	2.54±0.24	hypothetical protein	h
PG0947	PGA7_RS04815	PGN_1003		ND	2.18±0.06	hypothetical protein	d
PG0990	PGA7_RS04610	PGN_0964	*rpmI*	ND	2.22±0.13	50S ribosomal protein L35	p
PG1042	PGA7_RS04385	PGN_1310		ND	2.40±0.07	glycogen synthase	e
PG1075	PGA7_RS04245	PGN_1171		ND	2.31±0.20	succinyl-CoA--3-ketoacid-CoA transferase	e
PG1129	PGA7_RS04015	PGN_1226		ND	2.56±0.43	ribonucleoside-diphosphate reductase	n
PG1226	PGA7_RS03625	PGN_1364		ND	2.87±0.25	peptidyl-prolyl cis-trans isomerase	p
PG1251	PGA7_RS03525			ND	3.44±0.28	hypothetical protein	h
	PGA7_RS03495	PGN_1393		ND	2.25±0.20	integration host factor subunit beta	v
PG1613	PGA7_RS01785	PGN_0499	*mce*	ND	2.17±0.14	methylmalonyl-CoA epimerase	e
PG1691	PGA7_RS01450	PGN_0421		ND	3.05±0.05	DNA-binding protein	c
PG1729	PGA7_RS01275	PGN_0388	*tpx*	ND	5.02±0.80	thiol peroxidase	c
PG1987	PGA7_RS08865	PGN_1932	*cas10*	ND	2.68±0.47	type III-B CRISPR-associated protein Cas10/Cmr2	d
PG2006	PGA7_RS08950	PGN_1951		ND	6.22±1.40	hypothetical protein	h
PG2038	PGA7_RS09105	PGN_1985		-2.22±0.05	2.60±0.14	N-acetylmuramoyl-L-alanine amidase	l
PG2040	PGA7_RS09110	PGN_1986		-2.18±0.09	2.46±0.04	histidinol phosphate phosphatase	d
PG2154	PGA7_RS09655	PGN_0199		ND	2.32±0.05	hypothetical protein	h
PG2212		PGN_2076		ND	3.36±0.37	hypothetical protein	h
PG2213	PGA7_RS09935	PGN_2077		ND	3.84±0.28	nitrite reductase	h

a – amino acid biosynthesis; **b** – biosynthesis of cofactors/prosthetic groups; **c** – cellular processes; **d** – DNA metabolism; **e** – energy metabolism; **f** – fatty acid and phospholipid metabolism; **h** – hypothetical protein; **i** – central intermediary metabolism; **l** – cell envelope; **n** – purines, pyrimidines, nucleosides, and nucleotides; **o** – other; **p** – protein synthesis and protein fate; **r** – regulatory functions/signal transduction; **t** – transport and binding proteins; **v** – virulence. ND – changes in gene expression not detected.

TABLE S2 Analysis of gene expression in *P. gingivalis* grown for 20 hours in iron and heme-depleted conditions (DIP medium) in comparison to iron and heme-replete conditions (Hm medium) examined using microarray analysis – down-regulated genes.

Locus ID			Gene name	A7436	33277	Gene description	Category
W83	A7436	33277		Fold change (FC)			
PG0195	PGA7_RS00880	PGN_0302	*rbr*	-13.33±0.53	-61.16±8.75	rubrerythrin family protein	c
PG0303	PGA7_RS07655	PGN_1659		-2.60±0.07	-3.91±0.21	ferredoxin	e
PG0304	PGA7_RS07650	PGN_1658	*rsxC*	-3.84±0.30	-9.03±2.57	electron transporter RnfC	e
PG0305	PGA7_RS07645	PGN_1657		-4.69±0.54	-7.68±1.57	electron transport complex, RnfABCDGE type, D subunit	e
PG0306	PGA7_RS07640	PGN_1656		-4.66±0.17	-9.47±0.49	electron transporter RnfG	e
PG0307	PGA7_RS07635	PGN_1655		-4.08±0.26	-5.75±1.26	electron transporter RsxE	e
PG0308	PGA7_RS07630	PGN_1654		-3.86±0.34	-4.64±1.65	electron transport complex subunit RsxA	e
PG0451	PGA7_RS06990	PGN_1511		-3.10±0.90	-3.05±0.65	HlyC/CorC family transporter	v
PG0539	PGA7_RS06600	PGN_1431		-2.29±0.08	-5.98±1.57	RND transporter MFP subunit	c
PG0540	PGA7_RS06595	PGN_1430		-2.46±0.33	-6.85±1.62	multidrug transporter AcrB	c
PG0541	PGA7_RS06590	PGN_1429		-2.12±0.12	-3.90±0.42	hypothetical protein	h
PG0548	PGA7_RS06560	PGN_1418	*nifJ*	-13.53±2.63	-6.71±1.26	pyruvate:ferredoxin (flavodoxin) oxidoreductase	e
PG1171	PGA7_RS03835	PGN_1268		-3.99±0.97	-2.70±0.29	Fe-S oxidoreductase	e
PG1172	PGA7_RS03830	PGN_1269		-5.17±0.51	-4.05±0.94	4Fe-4S ferredoxin	e
PG1259	PGA7_RS03490	PGN_1395	*nrdG*	-3.04±0.47	-2.19±0.15	anaerobic ribonucleoside-triphosphate reductase activating protein	n
PG1260	PGA7_RS03485	PGN_1396		-3.63±0.33	-2.63±0.25	anaerobic ribonucleoside triphosphate reductase	n
PG1715	PGA7_RS01355	PGN_0400		-2.46±0.18	-3.36±0.41	outer membrane protein beta-barrel family	r
PG1727	PGA7_RS01285	PGN_0390		-2.33±0.07	-2.60±0.52	S1-like domain-containing RNA-binding protein	h
PG1779	PGA7_RS07925	PGN_1719		-2.62±0.36	-2.35±0.36	O-acetyl-ADP-ribose deacetylase	h
PG1809	PGA7_RS08070	PGN_1756		-6.17±0.37	-5.57±0.39	2-oxoglutarate ferredoxin oxidoreductase subunit gamma	e
PG1810	PGA7_RS08075	PGN_1755		-5.31±0.23	-5.83±0.69	2-oxoglutarate oxidoreductase	e
PG1812	PGA7_RS08080	PGN_1753	*vorB*	-4.44±0.37	-9.90±2.25	2-ketoisovalerate ferredoxin reductase	e
PG1820	PGA7_RS08115	PGN_1746	*nrfA*	-3.27±0.27	-2.18±0.17	ammonia-forming cytochrome c nitrite reductase subunit c552	e
PG1876	PGA7_RS08315	PGN_1803		-3.36±0.20	-2.45±0.16	glycerol acyltransferase	i
PG2065	PGA7_RS09230	PGN_2057	*rlmN*	-2.27±0.06	-2.28±0.04	23S rRNA (adenine[2503]-C[2])-methyltransferase	p
PG2071	PGA7_RS09260	PGN_2051		-2.25±0.14	-2.48±0.14	hypothetical protein	h
PG0091	PGA7_RS00410	PGN_2038		-2.21±0.23	ND	ABC transporter permease	t
PG0108	PGA7_RS00495	PGN_0224	*wecC*	-2.68±0.29	ND	UDP-N-acetyl-D-mannosamine dehydrogenase	l
PG0109	PGA7_RS00500			-2.58±0.41	ND	O-antigen ligase family protein	l
PG0218	PGA7_RS00975	PGN_0323		-2.06±0.06	ND	hypothetical protein	h
PG0256	PGA7_RS01130	PGN_0356		-3.60±2.70	ND	CvpA family protein/colicin V production protein	h
PG0272	PGA7_RS01195	PGN_0370	*gldE*	-2.29±0.15	ND	hemolysin	c
PG0285	PGA7_RS07740	PGN_1679		-5.59±0.75	ND	TolC family protein/Outer membrane efflux protein	c
PG0292	PGA7_RS07700	PGN_1671		-2.96±0.11	ND	chromate transporter	t

PG0320	PGA7_RS07575	PGN_1642		-2.16±0.04	ND	DUF1573 domain-containing protein	h
PG0321	PGA7_RS07570	PGN_1641	*meaB*	-2.30±0.09	ND	methylmalonyl CoA mutase-associated GTPase	t
PG0394	PGA7_RS07250	PGN_1571	*rpoB*	-2.24±0.10	ND	DNA-directed RNA polymerase subunit beta	r
PG0411	PGA7_RS07180	PGN_1556		-2.38±0.20	ND	hemagglutinin, putative	v
PG0413	PGA7_RS07170	PGN_1554		-2.18±0.13	ND	hypothetical protein	h
PG0441	PGA7_RS07040	PGN_1520		-2.15±0.18	ND	outer membrane protein beta-barrel family	r
PG0450	PGA7_RS06995	PGN_1512	*lptC*	-2.81±0.34	ND	LPS export ABC transporter periplasmic protein LptC	l
PG0511	PGA7_RS06750	PGN_1461		-2.31±0.36	ND	membrane protein/nucleoside recognition	e
PG0512	PGA7_RS06745	PGN_1460	*gmk*	-2.16±0.07	ND	guanylate kinase	n
PG0604	PGA7_RS06325	PGN_0647	*ispH*	-3.00±0.20	ND	4-hydroxy-3-methylbut-2-enyl diphosphate reductase	c
PG0623	PGA7_RS06255	PGN_0665	*tpiA*	-2.08±0.03	ND	triose-phosphate isomerase	e
PG0626	PGA7_RS06240			-2.96±0.17	ND	T9SS type A sorting domain-containing protein	o
PG0647	PGA7_RS06155	PGN_0686		-2.13±0.10	ND	iron ABC transporter permease/FecCD transport family	t
PG0679	PGA7_RS06015	PGN_0715		-11.45±0.21	ND	TolC family protein/outer membrane efflux protein	t
PG0684	PGA7_RS05990	PGN_0720		-3.63±0.38	ND	ABC transporter permease	t
PG0848	PGA7_RS05260			-2.40±0.17	ND	nucleotidyl transferase AbiEii/AbiGii toxin family protein	o
PG0849	PGA7_RS05255			-2.17±0.17	ND	DUF6088 family protein/transcriptional regulator	r
PG0862	PGA7_RS05195			-2.47±0.71	ND	Eco57I restriction-modification methylase domain-containing protein	d
PG0902	PGA7_RS05015	PGN_1039		-4.17±0.95	ND	GH92 family glycosyl hydrolase	l
PG0973	PGA7_RS04685	PGN_0980		-2.81±0.68	ND	alpha-mannosidase	l
PG0987	PGA7_RS04630	PGN_0968		-4.37±0.30	ND	DUF4252 domain-containing protein	h
PG1000	PGA7_RS04575			-2.33±0.55	ND	DNA alkylation repair protein	h
PG1002	PGA7_RS11735			-2.06±0.03	ND	hypothetical protein	h
PG1018	PGA7_RS04490			-4.47±0.88	ND	hypothetical protein	h
PG1030	PGA7_RS04445	PGN_1321		-2.48±0.03	ND	T9SS type A sorting domain-containing protein	o
PG1064	PGA7_RS04300	PGN_0906		-2.38±0.15	ND	dihydroorotate dehydrogenase electron transfer subunit	n
PG1065	PGA7_RS04295			-2.48±0.47	ND	dihydroorotate dehydrogenase	h
PG1066	PGA7_RS04290	PGN_1162		-2.21±0.03	ND	Coenzyme A transferase	h
PG1173	PGA7_RS03825	PGN_1270		-6.73±0.53	ND	LUD domain-containing protein	e
PG1208	PGA7_RS03710	PGN_0916	*dnaK*	-2.23±0.18	ND	molecular chaperone DnaK/Hsp70 protein	p
PG1330	PGA7_RS02925	PGN_1119	*mscL*	-2.06±0.03	ND	large-conductance mechanosensitive channel protein	o
PG1364	PGA7_RS02765	PGN_1151	*dxr*	-3.39±2.04	ND	1-deoxy-D-xylulose-5-phosphate reductoisomerase	b
PG1365	PGA7_RS02760	PGN_1152	*rimM*	-2.31±0.12	ND	ribosome maturation factor RimM	r
PG1366	PGA7_RS02755	PGN_1153	*murA*	-2.14±0.22	ND	UDP-N-acetylglucosamine 1-carboxyvinyltransferase	l
PG1389	PGA7_RS02660	PGN_0872		-2.15±0.07	ND	HU family DNA-binding protein	d
PG1417	PGA7_RS02545	PGN_0893		-2.86±0.25	ND	fumarate hydratase	e
PG1447	PGA7_RS05370			-2.90±0.93	ND	AraC family transcriptional regulator	r
PG1474	PGA7_RS02395			-3.96±0.66	ND	conjugal transfer protein TraO	o
PG1477	PGA7_RS02380			-2.07±0.09	ND	hypothetical protein	h
PG1481	PGA7_RS02360		*traG*	-6.32±2.38	ND	TraG family conjugative transposon ATPase	o

PG1493	PGA7_RS02300	PGN_0836		-2.69±0.34	ND	carboxypeptidase regulatory-like domain-containing protein	r
PG1501	PGA7_RS02280			-2.13±0.11	ND	TetR/AcrR family transcriptional regulator	r
PG1503	PGA7_RS02265		*lytB*	-3.75±0.58	ND	LytB protein	c
PG1507	PGA7_RS02245			-3.43±0.76	ND	hypothetical protein	h
PG1521	PGA7_RS02190			-2.58±0.22	ND	AMP-binding protein	r
PG1542	PGA7_RS02090	PGN_0567	*prtC*	-3.07±1.15	ND	U32 family peptidas/collagenase	p
PG1591	PGA7_RS01895			-2.29±0.16	ND	type I restriction enzyme HsdR N-terminal domain-containing protein	d
PG1626	PGA7_RS01725	PGN_0477		-5.47±0.62	ND	outer membrane protein transport protein	f
PG1682	PGA7_RS01490	PGN_0427		-2.23±0.10	ND	glycosyltransferase	e
PG1684	PGA7_RS01480			-2.58±0.32	ND	hypothetical protein	h
PG1768	PGA7_RS07875	PGN_1708		-3.19±0.05	ND	YifB family Mg chelatase-like AAA ATPase	h
PG1773	PGA7_RS07895	PGN_1713		-3.74±0.34	ND	phosphatase PAP2 family protein	h
PG1775	PGA7_RS07910	PGN_1716	*dnaJ*	-2.09±0.03	ND	molecular chaperone DnaJ	p
PG1777	PGA7_RS07915	PGN_1717		-2.06±0.04	ND	iron-sulfur cluster assembly protein	h
PG1778	PGA7_RS07920	PGN_1718		-2.38±0.07	ND	UDP-2,3-diacylglucosamine diphosphatase	l
PG1817	PGA7_RS08105	PGN_1748	*ccsA*	-4.48±0.72	ND	cytochrome *c* biogenesis protein CcsA	e
PG1819	PGA7_RS08110	PGN_1747		-2.16±0.08	ND	cytochrome *c* biogenesis protein ResB	e
PG1829	PGA7_RS08155	PGN_1738		-2.76±0.73	ND	long-chain-fatty-acid-CoA ligase	f
PG1836	PGA7_RS08175	PGN_1734	*nupG*	-3.25±0.43	ND	nucleoside permease/Nucleoside H+ symporter	t
PG1864	PGA7_RS10730			-2.76±0.65	ND	leucine-rich repeat protein	o
PG1880	PGA7_RS08335	PGN_1807		-2.25±0.05	ND	glycosyltransferase	h
PG1881	PGA7_RS08340	PGN_1808		-2.10±0.10	ND	family fimbria major subunit	v
PG1886	PGA7_RS08355	PGN_1813	*hflX*	-2.10±0.03	ND	GTPase HflX/50S ribosome-binding GTPase	h
PG2008	PGA7_RS08955			-2.72±0.45	ND	TonB-dependent receptor	t
PG2037	PGA7_RS09100	PGN_1984		-2.10±0.07	ND	hypothetical protein	h
PG2038	PGA7_RS09105	PGN_1985		-2.22±0.05	2.60±0.14	N-acetylmuramoyl-L-alanine amidase	l
PG2040	PGA7_RS09110	PGN_1986		-2.18±0.09	2.46±0.04	histidinol phosphate phosphatase	d
PG2054	PGA7_RS09170	PGN_1998		-2.18±0.05	ND	OmpA family protein	t
PG2100	PGA7_RS09405			-2.20±0.13	ND	T9SS type A sorting domain-containing protein	o
PG2101	PGA7_RS09410	PGN_0151		-3.15±0.50	ND	hypothetical protein	h
PG2130	PGA7_RS09545	PGN_0178		-2.27±0.06	ND	DUF3575 domain-containing protein	h
PG2131	PGA7_RS09550	PGN_0179		-3.20±0.28	ND	DUF3868 domain-containing protein	h
PG2132	PGA7_RS09555		*fimA*	-3.11±0.31	ND	fimbrial protein, fimbrillin	v
PG2133	PGA7_RS09560			-3.40±0.41	ND	FimB/Mfa2 family fimbrial subunit	v
PG2135	PGA7_RS09580			-3.18±1.03	ND	hypothetical protein	h
PG2149	PGA7_RS09635	PGN_0197	*porT*	-5.13±0.25	ND	PorT family protein, porin family protein	t
PG2150	PGA7_RS09640	PGN_0198		-3.59±0.36	ND	LysM peptidoglycan-binding domain-containing protein	h
PG2171	PGA7_RS09740	PGN_0125		-2.18±0.11	ND	2-hydroxyacid dehydrogenase family protein/D-isomer specific 2-hydroxyacid dehydrogenase	e
PG2185	PGA7_RS09800	PGN_0103		-3.15±0.39	ND	M56 family metallopeptidase	r

PG2186	PGA7_RS09805	PGN_0102		-2.15±0.09	ND	BlaI/MecI/CopY family transcriptional regulator	c
PG2208	PGA7_RS09900			-3.11±0.13	ND	GlsB/YeaQ/YmgE family stress response membrane protein, transglycosylase	h
PG2226	PGA7_RS10005	PGN_2090	*husB*	-3.74±1.53	ND	carboxypeptidase-like regulatory domain-containing protein	r
PG2227	PGA7_RS10010	PGN_2091	*husA*	-4.25±0.43	ND	putative hemophore-like protein	v
	PGA7_RS00605	PGN_0245		-2.37±0.36	ND	hypothetical protein	h
	PGA7_RS11740			-2.16±0.08	ND	hypothetical protein	h
	PGA7_RS03230	PGN_0066		-3.36±1.86	ND	DUF4133 domain-containing protein	h
	PGA7_RS03125			-3.33±1.61	ND	type IA DNA topoisomerase	d
	PGA7_RS03150			-3.44±0.42	ND	carboxypeptidase-like regulatory domain-containing protein	r
	PGA7_RS03165			-2.63±0.08	ND	hypothetical protein	h
	PGA7_RS03245	PGN_0063	*traJ*	-5.41±1.82	ND	conjugative transposon protein TraJ	o
	PGA7_RS03250	PGN_0062	*traK*	-3.46±0.65	ND	conjugative transposon protein TraK	o
	PGA7_RS03260	PGN_0060	*traM*	-5.80±0.82	ND	conjugative transposon protein TraM	o
	PGA7_RS03275			-2.43±0.28	ND	DNA primase	d
	PGA7_RS03325	PGN_0048		-4.30±0.54	ND	PcfK-like family protein	o
PG0093	PGA7_RS00420	PGN_2040		ND	-2.09±0.05	hemolysin secretion protein D	t
PG0538	PGA7_RS06605	PGN_1432		ND	-4.18±0.68	TolC family protein	c
PG0547	PGA7_RS06565	PGN_1419		ND	-3.81±0.36	ATPase AAA	h
PG0657	PGA7_RS06105	PGN_0695	*maf*	ND	-2.44±0.07	maf-like protein	c
PG0678	PGA7_RS06020	PGN_0714		ND	-2.21±0.11	pyrazinamidase	b
PG0986	PGA7_RS04635	PGN_0969		ND	-2.62±0.07	hypothetical protein	h
PG1058	PGA7_RS04320	PGN_1296		ND	-2.13±0.18	membrane protein	t
PG1248	PGA7_RS03540	PGN_1383		ND	-2.64±0.13	DNA alkylation repair protein	d
PG1270	PGA7_RS03430	PGN_1402		ND	-2.45±0.31	amidinotransferase	h
PG1393	PGA7_RS02645	PGN_0869	*mrdA*	ND	-2.30±0.01	penicillin-binding protein 2	l
PG1614	PGA7_RS01780	PGN_0498	*frdB*	ND	-2.48±0.06	succinate dehydrogenase	e
PG1618	PGA7_RS01765	PGN_0468		ND	-2.09±0.02	hypothetical protein	b
PG1813	PGA7_RS08085	PGN_1752		ND	-2.97±0.03	ferredoxin	e
PG1977	PGA7_RS08825	PGN_1907		ND	-2.60±0.06	hypothetical protein	h
PG2033	PGA7_RS09080	PGN_1980	*gltA*	ND	-2.87±0.39	dihydropyrimidine dehydrogenase subunit A	a
PG2049	PGA7_RS09150	PGN_1994		ND	-2.19±0.07	hypothetical protein	h
PG2159	PGA7_RS09680	PGN_0204	*hemG*	ND	-2.77±0.18	protoporphyrinogen oxidase	b

a – amino acid biosynthesis; **b** – biosynthesis of cofactors/prosthetic groups; **c** – cellular processes; **d** – DNA metabolism; **e** – energy metabolism; **f** – fatty acid and phospholipid metabolism; **h** – hypothetical protein; **i** – central intermediary metabolism; **l** – cell envelope; **n** – purines, pyrimidines, nucleosides, and nucleotides; **o** – other; **p** – protein synthesis and protein fate; **r** – regulatory functions/signal transduction; **t** – transport and binding proteins; **v** – virulence. ND – changes in gene expression not detected.

TABLE S3 Statistical analysis comparing the growth of A7436 and 33277 strains in liquid culture media containing iron and heme (iron and heme-replete conditions, Hm medium) or without heme and supplemented with the iron chelator 2,2-dipyridyl (iron and heme-depleted conditions, DIP medium) (left table). Recovery of bacteria after prior heme and iron starvation. Bacteria were cultured for 2 passages in the DIP medium and then transferred to the fresh DIP medium (DIP) without additives or supplemented with 7.7 µM heme (DIP+Hm) or 2 µM hemoglobin (DIP+Hb) (right table). Results present *P* values in certain time points. Statistically significant results ($P<0.05$) are marked in blue.

Time [h]	Hm medium	DIP medium
2	>0.9999	>0.9999
4	0.9962	>0.9999
6	0.2451	>0.9999
8	**<0.0001**	0.9633
10	**<0.0001**	0.2546
12	**<0.0001**	**0.0087**
14	**<0.0001**	**0.0003**
16	**<0.0001**	**<0.0001**
18	**<0.0001**	**0.0001**
20	**0.0002**	**0.0002**
22	**0.0009**	**0.0004**
24	**0.0023**	**0.0011**
26	**0.0074**	**0.0017**
28	**0.0118**	**0.0029**
30	**0.0196**	**0.0038**
32	**0.0444**	**0.0051**
34	0.0688	**0.0075**
36	0.1188	**0.015**
38	0.1678	**0.0251**
40	0.2451	**0.0404**
42	0.2351	0.0644
44	0.2853	0.0965
46	0.3218	0.1405
48	0.335	0.1936

Time [h]	DIP	DIP+Hm	DIP+Hb
2	>0.9999	>0.9999	>0.9999
4	>0.9999	>0.9999	>0.9999
6	>0.9999	>0.9999	>0.9999
8	>0.9999	>0.9999	>0.9999
10	0.999	>0.9999	>0.9999
12	0.948	0.9897	0.9736
14	0.8178	0.9396	0.5746
16	0.619	0.3897	0.2718
18	0.218	**0.0403**	**0.0403**
20	0.145	**0.0071**	**0.0009**
22	0.0994	**0.0005**	**<0.0001**
24	0.0704	**<0.0001**	**<0.0001**
26	**0.0133**	**<0.0001**	**<0.0001**
28	**0.0118**	**<0.0001**	**<0.0001**
30	**0.0276**	**<0.0001**	**<0.0001**
32	**0.0292**	**<0.0001**	**<0.0001**
34	**0.0191**	**<0.0001**	**<0.0001**
36	**0.025**	**<0.0001**	**<0.0001**
38	**0.029**	**<0.0001**	**<0.0001**
40	**0.0135**	**<0.0001**	**<0.0001**
42	**0.0054**	**<0.0001**	**<0.0001**
44	**0.0079**	**<0.0001**	**<0.0001**
46	**0.0069**	**<0.0001**	**<0.0001**
48	**0.001**	**<0.0001**	**<0.0001**

TABLE S4 Primers used in this study.

Primer name	5'→3' DNA sequence	Gene abbreviation, locus IDs (A7436, ATCC 33277, W83)	Description (reference)
HYq4_F	gcttcgaaatacgaaacgtg	*hmuY*, PGA7_RS02055, PGN_0558, PG1551	RT-qPCR analysis (1)
HYq4_R	tatatccgtctgtcggaacg		
F_hmuR	ctaccgacaccatcgtatcc	*hmuR*, PGA7_RS02050, PGN_0557, PG1552	RT-qPCR analysis (2)
R_hmuR	cattgagctgatctctggaac		
F_husA_qPCR	atcggctatgcgaagaagc	*husA*, PGA7_RS10010, PGN_2091, PG2227	RT-qPCR analysis (3)
F_husA_qPCR	gaagtaggctcgcggattag		
F_ihtB_qPCR	cagcaaacgatagagaaaggtg	*ihtB* (*fetB*), PGA7_RS06060, PGN_0705, PG0669	RT-qPCR analysis (this study)
R_ihtB_qPCR	ttcagttcggcaataacatca		
F_feoB_qPCR	cgggcacctattctctcctt	*feoB*, PGA7_RS04380, PGN_1309, PG1044	RT-qPCR analysis (2)
R_feoB_qPCR	tcggcttcgtccattagatt		
rtHagA2F	aggtgtacttggcattccgtc	*hagA*, PGA7_RS08180, PGN_1733, PG1837	RT-qPCR analysis (4)
rtHagA2R	cgtgtacgtgtagtcgttgga		
F_kgp_qPCR	gagtgtgggtgctaatgccg	*kgp*, PGA7_RS08195, PGN_1728, PG1844	RT-qPCR analysis (2)
R_kgp_qPCR	caccaatatgggtaatattgccg		
F_rgpA_qPCR	cgcttcccattctatcacgc	*rgpA*, PGA7_RS09040, PGN_1970, PG2024	RT-qPCR analysis (2)
R_rgpA_qPCR	cggatcttcgttacgcataatcat		
F_rgpB_qPCR	aatgataagccttatactgtagct	*rgpB*, PGA7_RS06770, PGN_1466, PG0506	RT-qPCR analysis (2)
R_rgpB_qPCR	gttttgtgcttcgaataccatgc		
16SrRNA-F	cttgacttcagtggcggcag	*16S rRNA*, PGA7_RS00460, PGN_r0001, PG16SA	RT-qPCR analysis (5)
16SrRNA-R	agggaagacggttttcacca		
F_rgpB	atgaaaaagaattttagcaggatcgtt	*rgpB*, PGA7_RS06770, PGN_1466, PG0506	Amplify the entire DNA sequence encoding RgpB protein (this study)
R_rgpB	ttacttcactataacctttctgtat		
PBM_0343	acaccatcaccaccatcacttaatcgagggaaggagcaac aacaaggatttggagaac	*ihtB* (*fetB*), PGA7_RS06060, PGN_0705, PG0669	Amplify the *ihtB* gene used to clone into XcmI and BamHI restriction sites of pTriEx-4 plasmid (this study)
PBM_0344	gagatctgagaattcggatccttagcgagcagaggtggctt		

1. Gmiterek A, Wojtowicz H, Mackiewicz P, Radwan-Oczko M, Kantorowicz M, Chomyszyn-Gajewska M, Fraszczak M, Bielecki M, Olczak M, Olczak T. 2013 The unique *hmuY* gene sequence as a specific marker of *Porphyromonas gingivalis*. PLoS ONE 8(7):e67719. doi: 10.1371/journal.pone.0067719.

2. Smiga M, Stepien P, Olczak M, Olczak T. 2019. PgFur participates differentially in expression of virulence factors in more virulent A7436 and less virulent ATCC 33277 *Porphyromonas gingivalis* strains. BMC Microbiol 19(1):127. doi: 10.1186/s12866-019-1511-x.
3. Smiga M, Slezak P, Wagner M, Olczak T. 2023. Interplay between *Porphyromonas gingivalis* hemophore-like protein HmuY and Kgp/RgpA gingipains plays a superior role in heme supply. Microbiol Spectr 11(2):e0459322. doi: 10.1128/spectrum.04593-22.
4. Ciuraszkiewicz J, Smiga M, Mackiewicz P, Gmiterek A, Bielecki M, Olczak M, Olczak T. 2014. Fur homolog regulates *Porphyromonas gingivalis* virulence under low-iron/heme conditions through a complex regulatory network. Mol Oral Microbiol 29:333-353. doi: 10.1111/omi.12077.
5. Maeda H, Fujimoto C, Haruki Y, Maeda T, Kokeguchi S, Petelin M, Arai H, Tanimoto I, Nishimura F, Takashiba S. 2003. Quantitative real-time PCR using TaqMan and SYBR Green for *Actinobacillus actinomycetemcomitans*, *Porphyromonas gingivalis*, *Prevotella intermedia*, *tetQ* gene and total bacteria. FEMS Immunol Med Microbiol 39:81-86. doi: 10.1016/S0928-8244(03)00224-4.
