# Supplementary material for: Comparative analysis of Porphyromonas gingivalis A7436 and ATCC 33277 strains reveals differences in the expression of heme acquisition systems
Source: Microbiol Spectr. 2024 Jan 30;12(3):e02865-23. doi: 10.1128/spectrum.02865-23 (PMC10913741; doi:10.1128/spectrum.02865-23)
Supplement: Fig. S1 and S2 — Supplementary data. [file spectrum.02865-23-s0002.pdf]

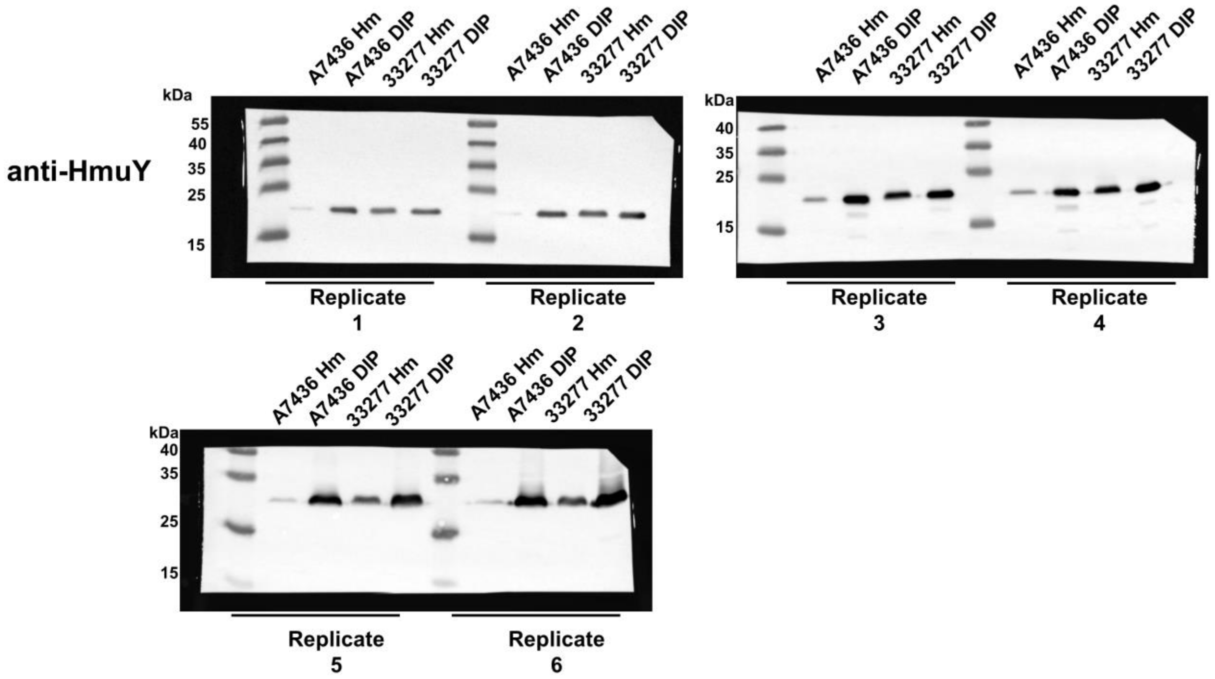
anti-HmuY
A7436 Hm
A7436 DIP
33277 Hm
33277 DIP
kDa
55
40
35
25
15
Replicate 1
Replicate 2
Replicate 3
Replicate 4
Replicate 5
Replicate 6

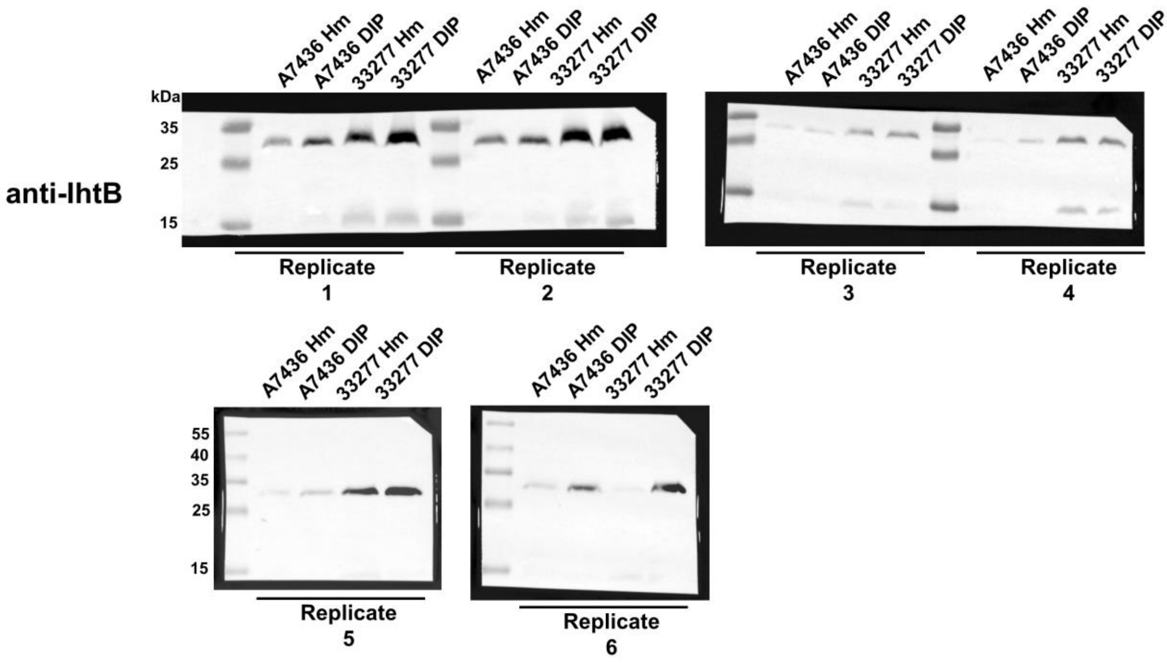
anti-IhtB
A7436 Hm
A7436 DIP
33277 Hm
33277 DIP
kDa
55
40
35
25
15
Replicate 1
Replicate 2
Replicate 3
Replicate 4
Replicate 5
Replicate 6

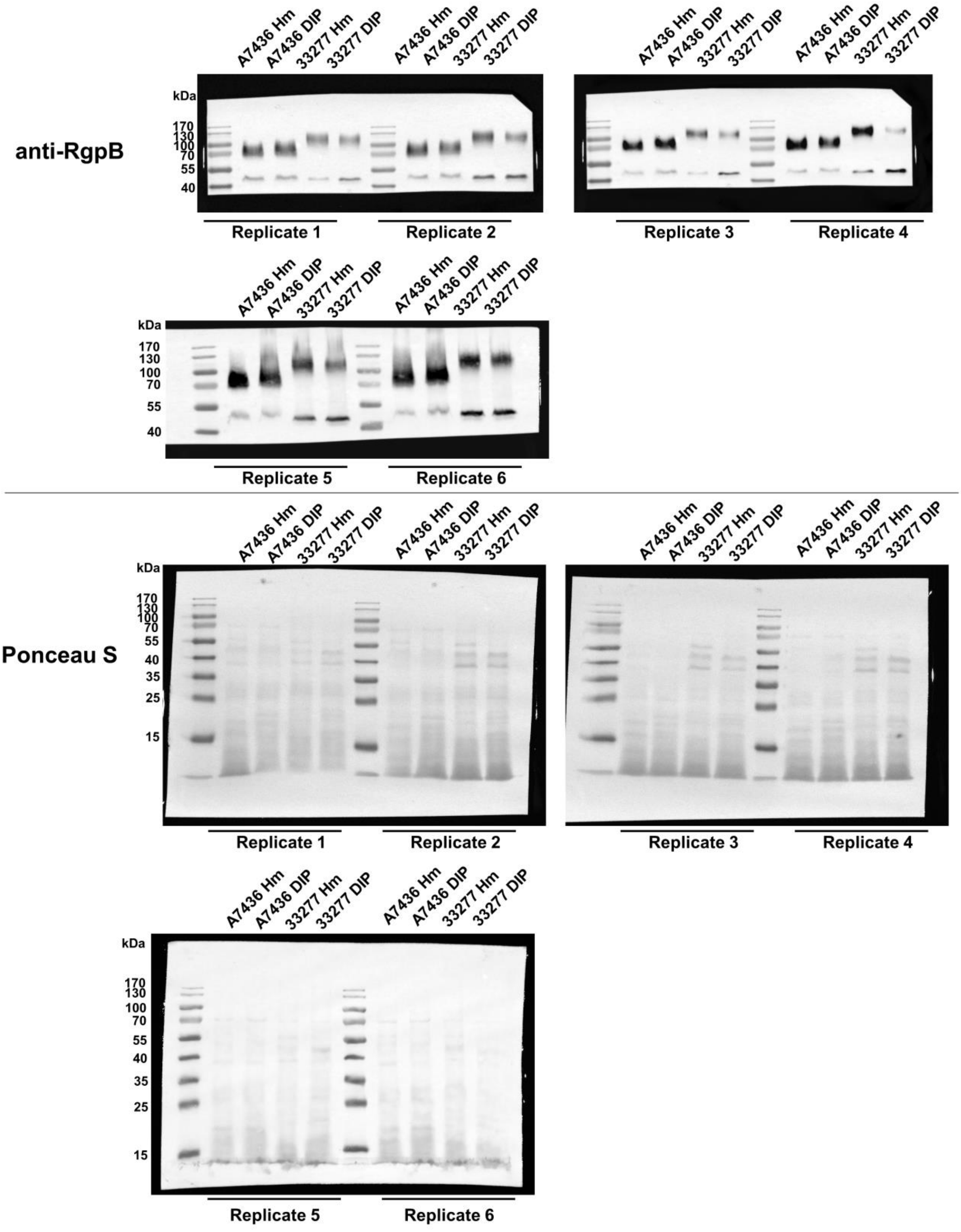

Fig S1 Supplemental material for Fig. 3C. Analysis of the production of the tested proteins using Western blotting with appropriate antibodies. Equal loading of proteins was visualized on nitrocellulose membranes by staining proteins with Ponceau S. All replicates used for densitometric analyses are shown. Hm, basal medium supplemented with 7.7 μM hemin (iron and heme-replete conditions). DIP, basal medium without added hemin and supplemented with 160 μM dipyridyl (iron and heme-deplete conditions).

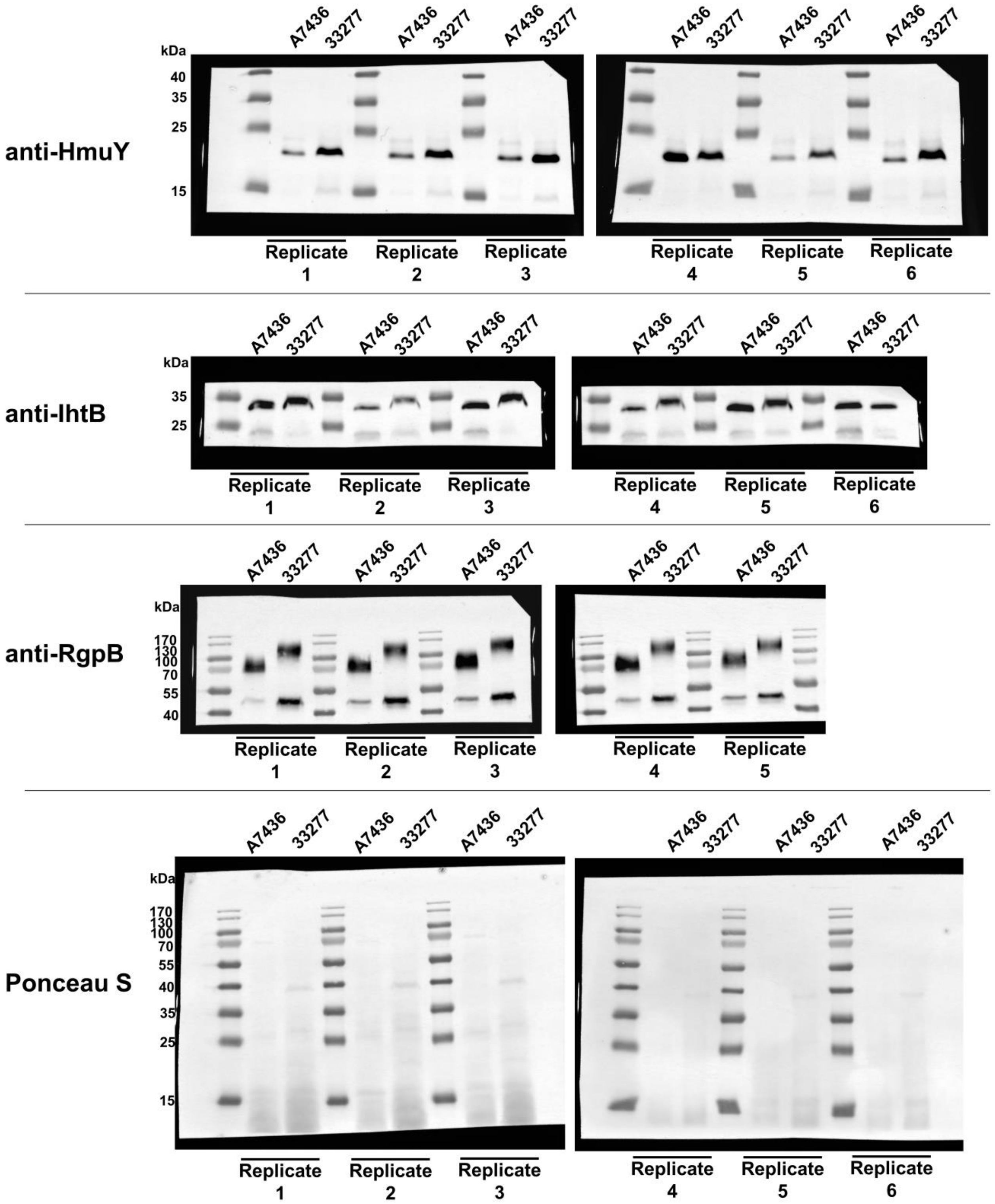

Fig S2 Supplemental material for Fig. 3D. Analysis of the production of the tested proteins using Western blotting with appropriate antibodies. Equal loading of proteins was visualized on nitrocellulose membranes by staining proteins with Ponceau S. All replicates used for densitometric analyses are shown.
